# Technological Advances in the Diagnosis of Cardiovascular Disease: A Public Health Strategy

**DOI:** 10.3390/ijerph21081083

**Published:** 2024-08-16

**Authors:** Maria Restrepo Tique, Oscar Araque, Luz Adriana Sanchez-Echeverri

**Affiliations:** 1 Facultad de Ingeniería, Universidad de Ibagué, Carrera 22 Calle 67, Ibagué 730002, Colombia; 2120161038@estudiantesunibague.edu.co; 2 Facultad de Ciencias Naturales y Matemáticas, Universidad de Ibagué, Carrera 22 Calle 67, Ibagué 730002, Colombia; luz.sanchez@unibague.edu.co

**Keywords:** cardiovascular diseases, artificial intelligence, machine learning, bibliometric analysis

## Abstract

This article reviews technological advances and global trends in the diagnosis, treatment, and monitoring of cardiovascular diseases. A bibliometric analysis was conducted using the SCOPUS database, following PRISMA-ScR guidelines, to identify relevant publications on technologies applied in the diagnosis and treatment of cardiovascular diseases. An increase in scientific output since 2018 was observed, reflecting a growing interest in the technologies available for the treatment of cardiovascular diseases, with terms such as “telemedicine”, “artificial intelligence”, “image analysis”, and “cardiovascular disease” standing out as some of the most commonly used terms in reference to CVDs. Significant trends were identified, such as the use of artificial intelligence in precision medicine and machine learning algorithms to analyse data and predict cardiovascular risk, as well as advances in image analysis and 3D printing. Highlighting the role of artificial intelligence in the diagnosis and continuous monitoring of cardiovascular diseases, showing its potential to improve prognosis and reduce the incidence of acute cardiovascular events, this study presents the integration of traditional cardiology methods with digital health technologies—through a transdisciplinary approach—as a new direction in cardiovascular health, emphasising individualised care and improved clinical outcomes. These advances have great potential to impact healthcare, and as this field expands, it is crucial to understand the current research landscape and direction in order to take advantage of each technological advancement for improving the diagnosis, treatment, and quality of life of cardiovascular patients. It is concluded that the integration of these technologies into clinical practice has important implications for public health. Early detection and personalised treatment of cardiovascular diseases (CVDs) can significantly reduce the morbidity and mortality associated with these diseases. In addition, the optimisation of public health resources through telemedicine and telecare can improve access to quality care. The implementation of these technologies can be a crucial step towards reducing the global burden of cardiovascular diseases.

## 1. Introduction

Cardiovascular diseases (CVDs) have gained global attention due to their increasing incidence. The risk of developing a cardiovascular disease increases with age, and according to statistics from the World Health Organization (WHO), one-third of the deaths associated with CVDs occur in people under 70 years of age [1].

Cardiovascular risk is defined as the probability of suffering a cardiovascular event in a given period of time; thus, CVDs have a multifactorial origin. Therefore, cardiovascular risk factors are divided into two main groups: modifiable factors (hypercholesterolaemia, smoking, diabetes, hypertension, obesity, and a sedentary lifestyle) and non-modifiable (age, sex, and family history) factors [2].

The World Health Organization (WHO) considers the detection and treatment of these diseases, as well as access to palliative care for those who need it, to be a priority. Basic high-impact interventions against non-communicable diseases (NCDs) can be delivered through a primary healthcare approach to strengthen early detection and timely treatment [3].

One of the main tools that can identify this risk and reduce the incidence of cardiovascular diseases is prevention [4]. The World Health Organization (WHO), in collaboration with experts from around the world, is facing the challenge of slowing or halting the progression of cardiovascular diseases. To address this major challenge comprehensively, it is recognised that it is important to focus on four key areas—prevention, diagnosis, treatment, and research [5]—in order to implement a solidly based global strategy to prevent and control non-communicable diseases (NCDs).

Cardiovascular diseases (CVDs) and public health have high health and economic burdens, and this has been studied by several different organisations and scientists.

The research in [6] identified the relationship connecting social isolation, low social support, and loneliness with health service utilisation and survival after a CVD event among people living in Australia and New Zealand; it showed that greater social support was consistently associated with better outcomes in terms of discharge destination, outpatient rehabilitation, re-hospitalisation, and survival at rates above 60%, with men being more likely to experience CVDs than women for a mean age varying between 46 and 82 years. Research has also been conducted showing that the prevention and treatment of cardiovascular diseases (CVDs) is related to the quality of life of patients in a way that is mainly associated with the surrounding environment: the research in [7] demonstrates that CVD incidence is related to the built environment and shows an upward trend with physical inactivity and poor environmental surroundings, as well as being affected by diet. Aspects such as access to transportation, place of residence, and greenery were the key themes of this research, showing that lower disease rates are related to decreased sedentary behaviour and that variability in the built environment is key to decreasing the prevalence of CVDs.

Building on the idea that the built environment plays a crucial role in the prevention and treatment of cardiovascular diseases, it is equally important to consider the transformative impact of technology on healthcare. The integration of digital advances in medical practice, known as eHealth, is not only improving the delivery of healthcare services but is also reshaping the traditional healthcare landscape [8]. This digital transformation is fostering the development of innovative, patient-centred care models that prioritise accessibility, quality, safety, and efficiency. As we continue to explore the interplay between the physical and digital realms, it is becoming clear that both the design of our environment and the use of technology are critical to addressing the challenges posed by cardiovascular diseases and improving patients’ overall quality of life.

Technology influences our daily lives and there is no doubt that advances in technology will have a significant impact on the way healthcare will be delivered in the future [9]. The irruption of technology in healthcare is an increasingly evident reality, driven by the rise of digital transformation. From this convergence between health and digital technology comes eHealth, which not only provides us with a toolbox that supports the development of innovative patient-centred care models, driving accessibility, quality, safety, and efficiency in all areas of healthcare, but which is also a cultural transformation of traditional healthcare, through disruptive technologies such as telemedicine, mobile health (mHealth), apps, artificial intelligence, sensors, and other devices [10].

Digital health technologies are rapidly changing the practice of medicine and redefining healthcare approaches [11]. The new wave of these digital advances that are making their way into clinical practice—including image and signal processing or advanced diagnostic imaging; artificial intelligence (AI) and big data; telemedicine; and new wearable devices [12]—is revolutionising the field of cardiology by improving diagnostic accuracy, optimising treatment, and enabling more efficient and personalised monitoring of cardiovascular patients. It is important to emphasise that the continuous technological progression experienced by the health sector requires the professional expertise in this area to be constantly updated.

New emerging technologies available and appropriate for the field of cardiovascular health can contribute to improving the quality of medical care by providing more accurate and timely information and allowing more effective treatments. Thus, to meet this need, it is important to develop technologies and platforms for telemonitoring vital signs and health-service-oriented strategies to establish and monitor the levels of risk associated with diseases such as cardiovascular diseases (CVDs), which will consequently reduce the number of physical visits to the clinic that a patient will need to make while ensuring greater control over the health of the patient [13].

One approach aiming to optimise diagnosis, risk prediction, prognosis, and intervention is precision medicine: by integrating large multimodal biomedical datasets incorporating data on different genes, functions, lifestyles, and environmental variations, precision medicine offers significant benefits over conventional medical methods through the use of these high-dimensional datasets to determine an individual’s health status, response to treatment, and prognosis [14]. Digital health is poised to transform healthcare and redefine personalised health as the adoption of new wearable, app-based, and AI technologies that develop new ways of data collection and controlled monitoring increases [11].

Modern medicine is characterised by continuous progress in digital innovations, and this is the result of convergence between potentially transformative technological developments and compelling applications that address healthcare needs and opportunities [15].

As the potential for technological advances in the diagnosis, treatment, and monitoring of heart disease continues to grow, it is essential to recognise the broader implications of these innovations. Not only do these technologies offer promising solutions to improve patient outcomes, but they also have important economic considerations. In this context, understanding the cost of treating cardiovascular diseases (CVDs) is crucial.

While few studies have delved into this aspect, the research in [16] has shed light on the economic burden in patients enrolled in a post-acute care programme for cerebrovascular disease (PAC-CVD). This study revealed that costs are substantially higher in the initial weeks (0 to 3) (mean = USD 2014.74) compared to later weeks (4 to 6) (mean = USD 1843.27), underlining the importance of early prevention strategies. Given these findings, it is clear that early intervention, together with a supportive environment and healthy lifestyle choices, is beneficial not only for patient health but also for managing the economic impact of CVDs. Therefore, this article aims to evaluate the existing literature through a structured systematic review, using biometric processes and PRISMA (Preferred Reporting Items for Systematic Reviews and Meta-Analyses) methodology, to identify global trends and advances in the use of these technologies for the diagnosis, treatment, and follow-up monitoring of cardiovascular diseases, with a strong interest in the potential economic benefits of early prevention and intervention.

## 2. Methods

**Figure 1:**
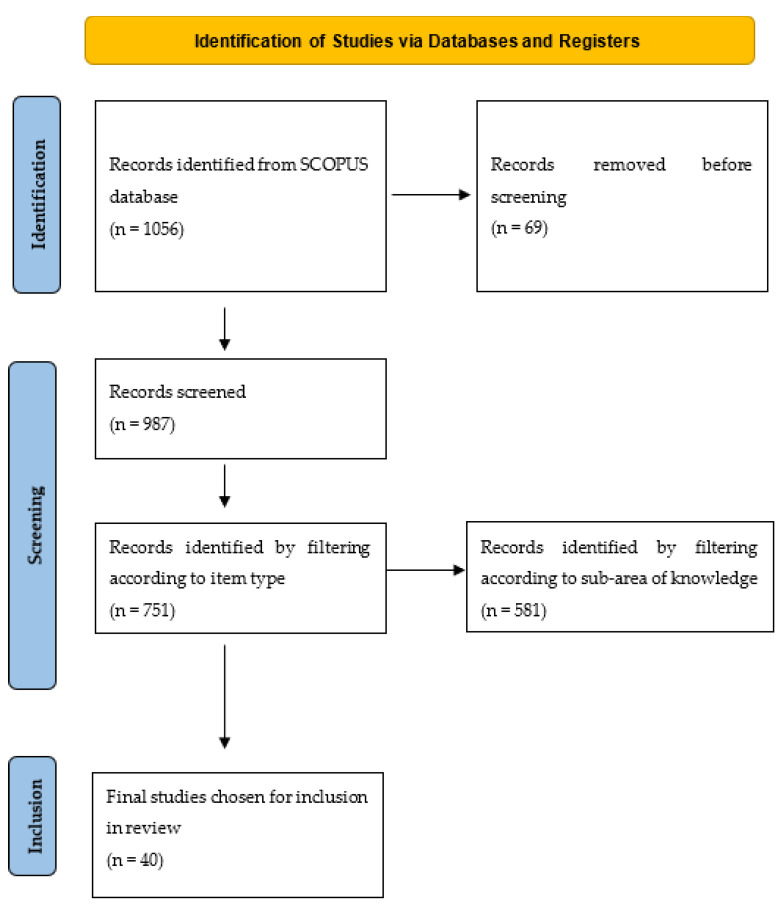
Flow diagram of the study selection process.

A literature search was conducted in a comprehensive database and a scoping review of the existing literature on technologies applied in the health sector for the diagnosis and treatment of patients with cardiovascular diseases was performed. We followed the steps recommended by the PRISMA-ScR (Preferred Reporting Items for Systematic Reviews and Meta-Analyses) guidelines [17] regarding the correct way to conduct systematic reviews, as shown in Figure 1, with an additional analysis established under the bibliometric parameters carried out using the Bibliometrix [18] package in R (4.4) statistical software, which allowed us to make some general conclusions after the first search was established.

### 2.1. Search Strategy

#### 2.1.1. Search Source

A bibliographic search was conducted in SCOPUS, which is a comprehensive database commonly used for bibliographic searches of abstracts and citations of scientific journal articles; the reference lists of the included studies were also examined to identify any additional relevant studies.

#### 2.1.2. Search Terms and Criteria

The initial search was set to range from 2010 to the present day (2023), combining main keywords such as “cardiovascular disease”, “technology”, “artificial intelligence”, “big data”, “digital stethoscope”, and “telemedicine” in the SCOPUS databases. This initial search yielded 1055 initial articles under the initial search equation TITLE-ABS-KEY (“cardiovascular disease” AND technology AND “artificial intelligence” OR “big data” OR “digital stethoscope” OR “telemedicine”). The following inclusion and exclusion criteria were then defined to refine the systematic search in the first instance.

Inclusion criteria:

- Publications within the last 13 years;
- Articles and reviews published in popular science journals.

Exclusion criteria:

- Publications prior to 2010;
- Books, book chapters, conferences, and other types of publications that are not published in indexed popular science journals;
- Research outside the field of health sciences and health technology.

## 3. Results

The search initially identified 1056 studies. Of these, 987 were selected after screening according to the publication year and this selection decreased to 751 papers after screening according to the paper type. An additional screening was then performed based on the area of study, selecting all those in the field of health sciences, cardiology, or engineering and digital technology aimed at the health sector, thus reducing the number of documents to 581. The same exercise was then performed excluding keywords that were not relevant to the topic of interest of this search, which reduced the selection to 40 articles. Thus, in total, 40 studies met the criteria and were included in the data extraction and synthesis process. The selection process is shown in Figure 1.

### 3.1. Scientific Production

**Figure 2:**
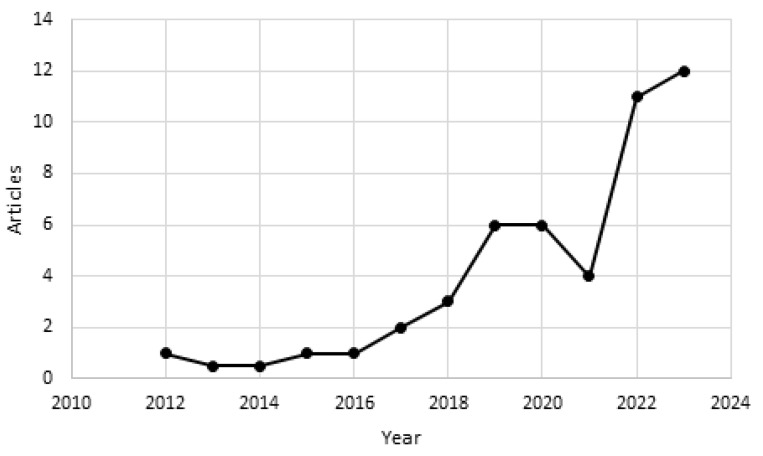
Annual scientific production.

As mentioned above, a total of 40 publications met the search criteria, and Figure 2 shows the distribution of these publications by year between 2010 and 2023. From 2018 onwards, the scientific production increased in the number of articles, reflecting a growing interest in the technologies available for the treatment of cardiovascular diseases. It can be said that in the period of 2021–2023, there was an increase in research on this topic, which we deduce could be because the end of the COVID-19 emergency gave rise to the need to design and develop technologies with great transformative potential and convincing applications that provide solutions to current needs. Figure 2 is based on data from the SCOPUS database, which includes research articles published in indexed scientific journals.

### 3.2. Most Relevant Journals

**Figure 3:**
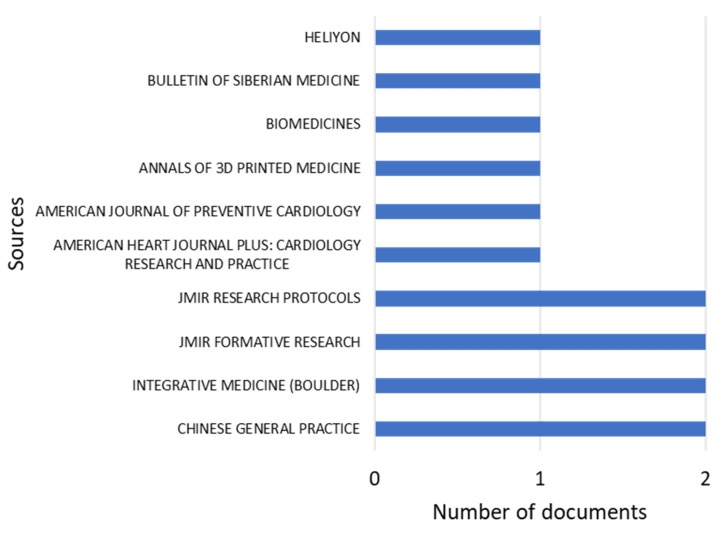
Most relevant journals from 2010 to 2023.

With regard to the most relevant journals that were found from the search criteria, Figure 3 shows the 10 most cited scientific journals in the research articles published in the last 13 years. Furthermore, these journals are mainly high-impact medical journals and leaders in the publication of cutting-edge scientific research. However, it can be observed that the number of publications per journal does not exceed two, which may infer that the other publications are distributed in a larger number of indexed journals that are not part of the top 10 obtained in this graph. It is worth mentioning that these journals are mostly from the USA, China, the Netherlands, and other developed countries.

### 3.3. Most Relevant Institutions

**Figure 4:**
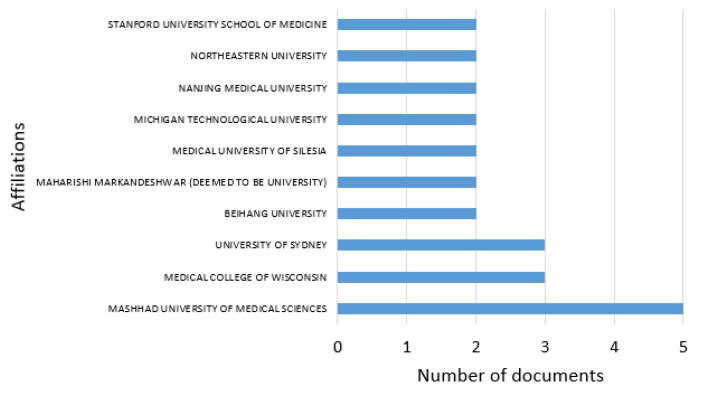
Most relevant affiliations producing research between 2010 and 2023.

In relation to the most relevant and outstanding higher-education institutions in terms of scientific production and important publications in indexed journals, Figure 4 shows 10 of the 127 institutions found in our selection process. The institutions shown in Figure 4 show a scientific production ranging between one and five publications; these high-level educational research centres are located in Iran, the United States, Australia, China, India, and Poland, with Mashhad University of Medical Sciences in Iran being the most relevant institution of those presented in Figure 4, with five published articles.

### 3.4. Cooperation between Countries

The network of cooperation between countries for the development of scientific research refers to collaborations and partnerships established between researchers, institutions, and nations with the aim of jointly developing research projects, sharing knowledge and resources, and publishing scientific articles in international journals. These networks are essential for addressing complex scientific challenges that require multidisciplinary expertise and large-scale collaboration [19].

Measuring the network of cooperation between countries for the construction of scientific articles can be carried out through several approaches, including the following:

Co-authorship analysis: This can include analysis of the institutional affiliation of authors and the frequency of collaboration between specific countries.

Joint scientific publications: This indicator can provide insight into the intensity and direction of international scientific cooperation.

Citations and cross-references: These can reflect the influence and impact of international cooperation on the generation of new knowledge.

Measuring these networks requires a multidisciplinary approach that integrates quantitative and qualitative data, allowing a deeper understanding of the patterns and effects of international scientific cooperation. In this context, a colour-coded visual representation of the collaboration network can be used, where the intensity of the colour indicates the level of influence of the originating authors on various aspects of the network, with a more intense colour indicating greater influence and a lower-intensity colour indicating the opposite.

**Figure 5:**
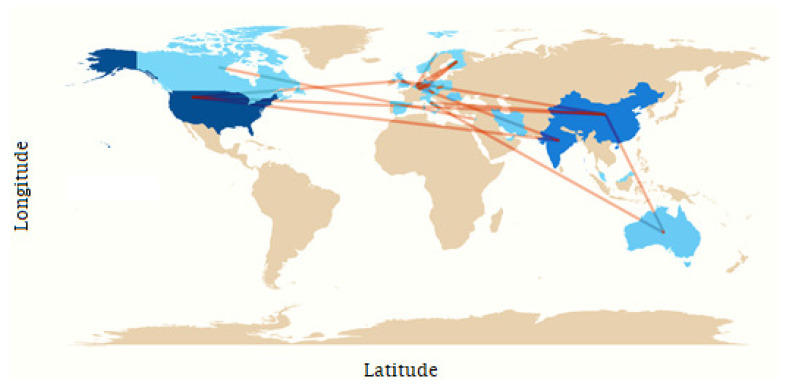
Map of collaboration between countries.

Turning to our analysis of the network of scientific collaboration, Figure 5 presents a world map highlighting the countries with the highest scientific output according to the dataset, using blue as an indicator. The shade of blue corresponds to the volume of scientific output, with darker shades representing higher levels of output. This visual representation clearly shows the United States, China, and India as the top countries in terms of scientific output. Furthermore, it reveals that four of the five continents are involved in scientific collaboration, and each represented country has at least one interaction with another country, underlining the global nature of scientific cooperation.

### 3.5. Keyword Analysis

The analysis of a thematic keyword map involves interpreting the visual representation of the data to understand the patterns and relationships between keywords. It involves a keyword co-occurrence analysis to identify the frequency and strength of associations between keywords, clusters keywords based on their co-occurrence patterns, with the purpose of identifying themes or topics within the dataset [20].

It creates a thematic map where each cluster is represented by a different colour, these colours are usually assigned to groups based on their properties or the results of the clustering algorithm, e.g., lighter shades represent the lower frequency or importance of keywords within the group versus darker shades.

**Figure 6:**
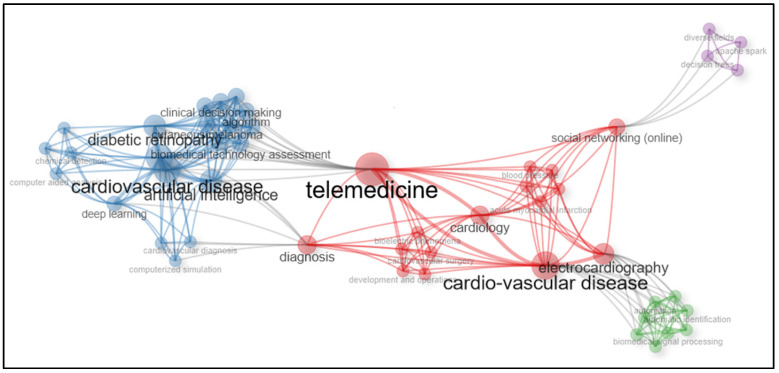
Thematic map of the keyword co-occurrence network.

The keyword co-occurrence network is shown in Figure 6, where the size and colour of the node represent the number of keywords and cluster, respectively. In this case, Figure 6 is a broad network of the keywords that were found in the selected papers, it is important to note that the colours indicate keywords in different topics.

**Figure 7:**
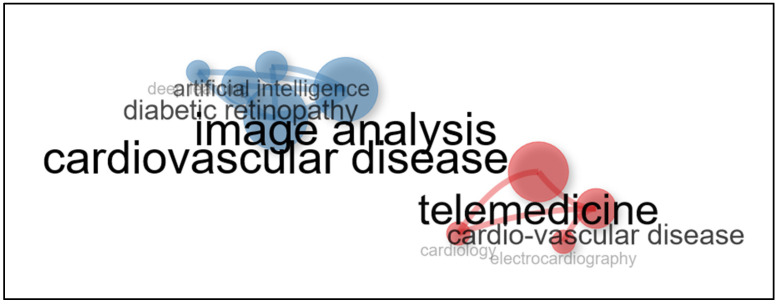
Keyword co-occurrence network.

Below, Figure 7 shows in more detail the five largest nodes, which are telemedicine, artificial intelligence, image analysis, diabetic retinopathy, and cardiovascular disease (or “cardio-vascular disease”); these keywords co-occur frequently in CVD-related studies, reflecting the increasing integration of advanced technologies in the diagnosis, treatment, and monitoring of these diseases. The combination of these technologies enables early detection, more efficient management, and improved quality of life for patients, highlighting the importance of technological innovation in the fight against CVD.

In the field of public health, cardiovascular diseases (CVDs) represent a serious problem affecting millions of people worldwide. Bibliometric analysis reveals that CVD research has experienced significant growth in recent decades, with an increasing focus on the development of innovative technologies for treatment and early detection. These technologies include medical image analysis, telemedicine, and artificial intelligence (AI), which are transforming the ways in which CVD prevention and management is approached. The integration of these tools not only improves clinical outcomes but also optimises public health resources, underlining the importance of technological innovation in the fight against CVDs.

### 3.6. Medical Technology

Diagnosis is made through an inferential process based on information provided by a pre-existing set of clinical symptoms, used by physicians for the purpose of identifying a specific disease affecting a patient.

Most cardiac diseases are associated and related to heart sounds. Cardiac auscultation, defined as listening to and interpreting the heart sound (HS), has been a very important method for early diagnosis of cardiac diseases by capturing abnormal HSs [21]. Auscultation of the heart has traditionally been limited by three factors. Firstly, HS contains a mixture of high-frequency (HF) and low-frequency (LF) acoustic signals with low amplitude. Secondly, HS data recorded with a stethoscope are often corrupted by noise, which can impede the accurate and effective diagnosis of heart disease. Thirdly, the interpretation of HS is highly subjective and depends largely on the experience, skills, and hearing ability of the clinician [21].

Digital signal processing (DSP)-based techniques can be used to combat the fact that many cardiac sounds associated with defects remain subtle and difficult to detect and distinguish from similar sounds without underlying pathology. Some of the same technological advances that have supported DSP-oriented methods have also facilitated the development of increasingly sophisticated models of the heart, from relatively simple models centred on sound formation to full three-dimensional structural models [22].

Recently, methodologies linked to advances in the field of electronic stethoscopy have been developed to process the auscultatory signals of the heart in order to analyse and elucidate the resulting sounds to make diagnoses based on quantifiable medical assessments. Importantly, due to advancements in sensor technologies, digital signal processing techniques, and the digital sound transmission capabilities of currently emerging stethoscopes, the emergence and further development of computer-aided electronic stethoscopy may become a major step forward from traditional acoustic equipment. This allows physicians not only to hear but also to see, record, and transfer auscultatory data from the heart, thus enabling a more complete and accurate diagnostic assessment [21].

CVD imaging is mainly based on detection methods related to cardiac ultrasound, cardiovascular (CV) angiography, CV MRI, and computed tomography [23]. CV image analysis has evolved with the use of deep learning (DL) techniques; thus, the diagnostic methodology under image analysis in cardiovascular diseases combines image acquisition and processing, feature extraction, classification, and diagnosis, with the aim of improving accuracy and efficiency in the detection and treatment of cardiac conditions.

Artificial intelligence (AI)-driven medical technologies are rapidly evolving into solutions applicable to clinical practice. Artificial intelligence (AI) is an exciting new field in cardiovascular diseases that is revolutionising the medical field [24]. AI can effectively help physicians diagnose cardiovascular diseases and perform continuous monitoring for early detection and treatment, thereby reducing the occurrence of acute cardiovascular events and improving prognosis [25].

Currently, deep learning (DL) is a field in machine learning (ML) research that refers to computational algorithms that iteratively improve their ability to recognise patterns in data [26] and can handle increasing amounts of information provided by handheld devices, smartphones, and other mobile monitoring sensors in different areas of medicine [24].

Deep learning (DL) is a widely adopted form of AI in medical image processing with many frameworks to choose from, such as convolutional neural networks (CNNs), recurrent neural networks (RNNs), stacked automatic encoders (SAEs), and deep belief networks (DBNs) [27]. Furthermore, medical imaging, computed tomography (CT), magnetic resonance imaging (MRI), and ultrasound imaging are other useful imaging modalities [28] that have become an integral part of the diagnosis and treatment of cardiovascular diseases and are becoming increasingly important. In the field of CV medical image analysis, three categories are used, namely classification, detection, and segmentation. This structured approach makes it possible to comprehensively address various dimensions of visual information in the medical field. In parallel, with the rapid development of medical imaging technology, medical image analysis has entered the era of “big data” in terms of the methods used to extract useful knowledge from a large amount of CV medical images and provide a more accurate basis for clinical diagnosis [23].

In recent years, AI has been widely explored in the detection of eye diseases from colour fundus photography (CFP), including diabetic retinopathy and glaucoma. Based on the improved measurement of retinal vascular features and the advantages of AI in detecting small changes in blood vessels, AI has begun to be applied to predict CVDs from eye images, and some models have shown comparable performance in CVD stratification or risk prediction compared to conventional methods [29].

To conduct a successful study, it is essential to have certain variables or prior information that allows artificial intelligence (AI) algorithms to effectively analyse ocular characteristics and their relationship to cardiovascular diseases. Demographic and clinical data such as age, gender, family history of CVDs, and information from the patient’s medical history showing current health conditions, medical treatments, and additional risk factors can be crucial for a complete cardiovascular risk assessment. Risk detection through ocular imaging is achieved through detailed analysis of the vascular structure and biomarkers present in the image, using AI algorithms to identify patterns and correlations that may indicate the likelihood of a cardiovascular disease being present. This innovative approach enables non-invasive and early assessment of cardiovascular risk, which can lead to preventive and personalised intervention to improve patient outcomes.

Moreover, the use of big data allows healthcare providers and administrators to drill down into their patients’ histories and enhance the care that they provide to them. Electronic health records (EHRs), medical imaging, genetic sequencing, payment records, pharmaceutical research, wearable devices, and medical devices, to name a few, are examples of applications of big data in healthcare [30].

Continuing with emerging technologies, with the advent of digital health technologies, it is now possible to monitor a patient’s health continuously. Wearable devices, including smartwatches and physical activity trackers, can measure a variety of physiological parameters such as heart rate, blood pressure, sleep patterns, and physical activity levels, allowing early detection of abnormalities that could indicate an impending cardiovascular event [31]. In particular, some experts have proposed the use of a transdisciplinary cardiovascular health concept, which is an innovative approach that integrates multiple disciplines and technologies to comprehensively address heart health and presents two fundamental concepts: digitalomics and digital intervention. Digitalomics refers to the use of digital technologies to collect and analyse large amounts of biological data, enabling a better understanding of cardiovascular physiology and pathology. Furthermore, digital interventions, such as wearable devices and mobile apps, enable continuous monitoring of cardiovascular health and personalisation of treatments [31]. This approach aims to transform the ways in which cardiovascular health is approached and is opening up new possibilities for the future of cardiology.

In relation to cardiology, 3D printing plays an important role in the advancement of cardiovascular treatments. By developing 3D-printed models of various materials and increasing complexity, a better understanding of the anatomy of the heart and its relationship to pathologies can be achieved [28].

Three-dimensional (3D) printing has proven to be a revolutionary and promising tool in many medical applications in the field of CVDs by enabling the creation of customised and accurate models that reproduce a patient’s anatomy accurately. These models are used to improve pre-operative planning, medical education, patient communication, and simulations of complex procedures. Furthermore, rapid prototyping has opened up new possibilities in the development of customised medical devices, such as bioabsorbable coronary stents, and in research on bioprinting cardiovascular tissues. These potential advances in 3D printing technology have a significant impact on cardiovascular medicine by improving the understanding of heart disease, optimising treatments, and personalising patient care, ultimately leading to better clinical outcomes and more effective care [32].

On the other hand, the World Health Organization (WHO) recently announced their Global Strategy on Digital Health 2020–2024, with the vision of “improving health for everyone, everywhere, by accelerating the adoption of appropriate digital health” [33]. It is thus important to mention that digital health has many facets, including eHealth, mHealth, medical informatics, and telemedicine [34]. Telemedicine technology provides healthcare services in situations where distance is a factor affecting a patient’s access to certain care. Specialised personnel use telecommunication technologies to exchange valid information for the diagnosis, treatment, and prevention of illness and injury, as well as for the continuing health education of those in need [35].

For cardiac diagnostic telemedicine systems, acoustic cardiography is increasingly being used in the diagnosis of heart disease. This type of examination collects acoustic signals using a specialised device consisting of a digital condenser microphone and a device mounted on the patient’s body [36], such as the digital or traditional stethoscope equipment mentioned above. To establish a good diagnosis, it is important that a diagnostic programme takes into account the important indicators from which the visualisation of biomedical signals will result. The resulting images provide graphs of the behaviour of the heart according to heart sounds, respiratory sounds, and speech signals that characterise the current functioning of a person’s heart, lungs, and vocal tract. This medical practice can contribute to the early diagnosis of ventricular tachycardia, ventricular fibrillation, myocardial ischaemic lesions, and myocardial infarction [36].

## 4. Discussion

In this section, we discuss the findings obtained through our bibliometric analysis of the literature on cardiovascular diseases (CVDs) and the technologies used for their treatment and early detection. Our literature review has highlighted the growing importance of artificial intelligence (AI) in precision medicine, particularly in the context of CVDs. AI models are fundamental for the implementation of P4 (predictive, preventive, personalised, and participatory) medicine, providing new tools for the diagnosis, prediction, and monitoring of these diseases [31].

Several studies have shown that machine learning (ML) and deep learning (DL) are able to analyse large volumes of clinical and imaging data, identifying patterns and predicting risks with unprecedented accuracy [37]. This enables personalisation of treatments and improved clinical outcomes. For example, AI can analyse medical imaging data to detect cardiac abnormalities that might go unnoticed by the human eye, particularly those associated with an increased risk of developing a CVD. These advances enable earlier and more effective intervention, reducing the burden of disease in the global population.

Telemedicine and telehealth have emerged as innovative approaches that enable continuous patient monitoring, facilitating digital interventions that optimise cardiovascular health. Using wearable devices and mobile apps, patients can monitor their vital signs in real time, and clinicians can access this information remotely to make informed clinical decisions [38]. This not only improves patients’ treatment adherence and quality of life, but also reduces the need for frequent hospital visits, optimising public health resources.

The use of interactive 3D visualisation (i3DV) and the integration of multimodality imaging in cardiology represent significant advances in physicians’ diagnostic and therapeutic capabilities. These technologies enable a deeper understanding of complex cardiac abnormalities, improving diagnostic accuracy and treatment efficacy. i3DV allows physicians to visualise cardiac structures in three dimensions, facilitating surgical planning and a deep understanding of the heart’s anatomy and function [28]. In addition, the integration of multimodality imaging, such as the fusion of magnetic resonance imaging (MRI) and computed tomography (CT), provides a more complete view of cardiovascular diseases, which can lead to more accurate diagnoses and more effective treatments.

The integration of these technologies into clinical practice has important implications for public health. Early detection and personalised treatment of CVDs can significantly reduce the morbidity and mortality associated with these diseases [39]. In addition, the optimisation of public health resources through telemedicine and telehealth can improve access to quality healthcare, especially in remote or resource-poor regions. The implementation of these technologies can be a crucial step towards reducing the global burden of cardiovascular diseases.

However, despite significant advances, the implementation of these technologies faces several challenges. Privacy and data security are major concerns, especially with the increased use of wearable devices and mobile applications. In addition, challenges related to interoperability between different systems and data platforms can be an obstacle to the widespread adoption of these technologies. However, these challenges also present opportunities for innovation and collaboration between different actors in the healthcare ecosystem.

## 5. Conclusions

This article has aimed to identify global trends and advances in the use of the aforementioned technologies for the diagnosis, treatment, and monitoring of cardiovascular diseases. A literature review was conducted in the SCOPUS database and this highlighted an increasing use of precision medicine and machine learning algorithms to analyse data and identify relevant patterns to help predict cardiovascular risks that patients may present. The findings in the literature suggest that artificial intelligence is enabling the development of a new field in medicine that holds promise for modern areas such as precision medicine and telemedicine. On the other hand, in the last two decades, the use of AI, specifically in the area of machine learning (ML), is growing in the field of cardiology. DL methods in the field of computer vision have become increasingly mature and DL algorithms and big data have fully demonstrated the potential to solve the dilemma faced by traditional CV image analysis methods. These image analyses have become an integral part of CVD diagnosis and treatment and are increasingly important.

It is also worth mentioning that advances in 3D printing have proven it to be a promising technology, with interesting potential applications in medicine and the treatment of cardiovascular diseases, in particular in stent technology, allowing it to become an innovative breakthrough for improving patient outcomes.

On the other hand, telemedicine, as an innovation in healthcare delivery, brings with it a number of substantial benefits. One of these is that it significantly expands access to healthcare by overcoming geographical barriers and providing health services to remote or under-resourced areas, thereby reducing waiting times and improving efficiency in service delivery. Telemedicine facilitates continuous monitoring of chronic patients and long-term disease management, improving quality of life and reducing unnecessary hospitalisations. In addition, by fostering communication between healthcare professionals and patients and providing an agile response in emergency situations, telemedicine demonstrates its valuable role in improving the accessibility, efficiency, and quality of healthcare.

These advances have great potential to impact healthcare, and as this field of interest expands, it is crucial to understand the current research landscape and direction in order to take advantage of every technological advancement to enhance the diagnosis, treatment, and quality of life of cardiovascular patients.

In conclusion, the integration of these technologies into clinical practice has important implications for public health. Early detection and personalised treatment of CVDs can significantly reduce the morbidity and mortality associated with these diseases. In addition, the optimisation of public health resources through telemedicine and telehealth can improve patients’ access to quality healthcare. The implementation of these technologies can serve as a crucial step towards reducing the global burden of cardiovascular diseases, with AI-based precision medicine and digital interventions offering new opportunities for CVD prevention and treatment.

## Data Availability

The data presented in this study are available in Supplementary Materials.

## Supplementary Materials

The following supporting information can be downloaded at: https://www.mdpi.com/article/10.3390/ijerph21081083/s1.
