# Supplementary material for: Technological Advances in the Diagnosis of Cardiovascular Disease: A Public Health Strategy
Source: Int J Environ Res Public Health. 2024 Aug 16;21(8):1083. doi: 10.3390/ijerph21081083 (PMC11354672; doi:10.3390/ijerph21081083)
Supplement: Supplementary file 1 [file ijerph-21-01083-s001.zip › ijerph-3136841-supplementary.pdf]

Supplementary Materials

**article**	**Li2022**
abstract	Cardiovascular disease (CVD) is the most common cause of morbidity and mortality worldwide, and early accurate diagnosis is the key point for improving and optimizing the prognosis of CVD. Recent progress in artificial intelligence (AI), especially machine learning (ML) technology, makes it possible to predict CVD. In this review, we first briefly introduced the overview development of artificial intelligence. Then we summarized some ML applications in cardiovascular diseases, including ML–based models to directly predict CVD based on risk factors or medical imaging findings and the ML–based hemodynamics with vascular geometries, equations, and methods for indirect assessment of CVD. We also discussed case studies where ML could be used as the surrogate for computational fluid dynamics in data–driven models and physics–driven models. ML models could be a surrogate for computational fluid dynamics, accelerate the process of disease prediction, and reduce manual intervention. Lastly, we briefly summarized the research difficulties and prospected the future development of AI technology in cardiovascular diseases.
author	Li, Xiaoyin and Liu, Xiao and Deng, Xiaoyan and Fan, Yubo
doi	10.3390/biomedicines10092157
issn	22279059
journal	Biomedicines
number	9
pages	1--21
title	Interplay between Artificial Intelligence and Biomechanics Modeling in the Cardiovascular Disease Prediction
volume	10
year	2022
**misc**	**OMS2022**
author	OMS
title	Enfermedades no transmisibles
year	2022
**article**	**Reed2004**
abstract	Heart auscultation (the interpretation by a physician of heart sounds) is a fundamental component of cardiac diagnosis. It is, however, a difficult skill to acquire. In this work, we develop a simple model for the production of heart sounds, and demonstrate its utility in identifying features useful in diagnosis. We then present a prototype system intended to aid in heart sound analysis. Based on a wavelet decomposition of the sounds and a neural network-based classifier, heart sounds are associated with likely underlying pathologies. Preliminary results promise a system that is both

	accurate and robust, while remaining simple enough to be implemented at low cost. © 2004 Elsevier B.V. All rights reserved.
author	Reed, Todd R. and Reed, Nancy E. and Fritzson, Peter
doi	10.1016/j.simpat.2003.11.005
issn	1569190X
journal	Simulation Modelling Practice and Theory
number	2
pages	129--146
title	Heart sound analysis for symptom detection and computer-aided diagnosis
volume	12
year	2004
**article**	**Moher2009**
author	Moher, David and Liberati, Alessandro and Tetzlaff, Jennifer and Altman, Douglas G. and Antes, Gerd and Atkins, David and Barbour, Virginia and Barrowman, Nick and Berlin, Jesse A. and Clark, Jocalyn and Clarke, Mike and Cook, Deborah and D'Amico, Roberto and Deeks, Jonathan J. and Devereaux, P. J. and Dickersin, Kay and Egger, Matthias and Ernst, Edzard and Gøtzsche, Peter C. and Grimshaw, Jeremy and Guyatt, Gordon and Higgins, Julian and Ioannidis, John P.A. and Kleijnen, Jos and Lang, Tom and Magrini, Nicola and McNamee, David and Moja, Lorenzo and Mulrow, Cynthia and Napoli, Maryann and Oxman, Andy and Pham, Bá and Rennie, Drummond and Sampson, Margaret and Schulz, Kenneth F. and Shekelle, Paul G. and Tovey, David and Tugwell, Peter
doi	10.1371/journal.pmed.1000097
isbn	2006062298
issn	15491676
journal	PLoS Medicine
number	7
pmid	19621072
title	Preferred reporting items for systematic reviews and meta-analyses: The PRISMA statement
volume	6
year	2009

article	Baladron2021
abstract	Technological progress in medicine is constantly garnering pace, requiring that physicians constantly update their knowledge. The new wave of technologies breaking through into clinical practice includes the following: a) mHealth, which allows constant monitoring of biological parameters, anytime, anyplace, of hundreds of patients at the same time; b) artificial intelligence, which, powered by new deep learning techniques, are starting to beat human experts at their own game: diagnosis by imaging or electrocardiography; c) 3-dimensional printing, which may lead to patient-specific prostheses; d) systems medicine, which has arisen from big data, and which will open the way to personalized medicine by bringing together genetic, epigenetic, environmental, clinical and social data into complex integral mathematical models to design highly personalized therapies. This state-of-the-art review aims to summarize in a single document the most recent and most important technological trends that are being applied to cardiology, and to provide an overall view that will allow readers to discern at a glance the direction of cardiology in the next few years.
author	Baladrón, Carlos and {Gómez de Diego}, José Juan and Amat-Santos, Ignacio J.
doi	10.1016/j.recesp.2020.06.017
issn	15792242
journal	Revista Espanola de Cardiologia
number	1
pages	81--89
pmid	33008773
title	Big data and new information technology: what cardiologists need to know
volume	74
year	2021
**article**	**Garavand2022a**
abstract	Background: Telemedicine is vital technology to deliver health services at a distance by health professionals, especially physicians, who are key players in Community health. Given the important role of telemedicine in improving health care, especially in the COVID-19 epidemic, an examination of behavioral barriers and not using this technology among physicians can be important. Objectives: The aim of our systematic review is to identify the behavioral factors influencing the acceptance of telemedicine technology among physicians in different contexts. Methods: A literature search was conducted according to the PRISMA guidelines. The search was conducted without any time limitations up to the Dec of 2020 in Web of Science, PubMed, Scopus, and Embase scientific databases; by applying keywords. The article selection was made based on inclusion (telemedicine among physicians, using the acceptance behavioral theories), and exclusion (physicians not the end-users of technology, it is not about acceptance of technology) criteria by two authors independently. Data was gathered using a data extraction form, and the results were reported in ta-

	bles and figures based on the study objectives. Results: From all the retrieved studies, 37 articles were included based on the inclusion and exclusion criteria. The United States and Spain have the most conducted studies about the acceptance of telemedicine from the physicians' point of view. The study results showed that the Technology Acceptance Model (TAM) and extended TAM model have the highest frequency. The main factors affecting the acceptance and use of telemedicine were perceived usefulness, attitude to use, compatibility, perceived ease of use, self-efficacy, subjective norms, perceived behavioral control, and facilitating condition. Conclusions: Identifying the most important factors that affect the acceptance of telemedicine from physicians' perspectives, as a key player in telemedicine projects, can help managers and policymakers make the right decisions about implementation of telemedicine successfully, especially in the initial phases. Future studies can also evaluate the aggregation of factors identified in this paper.
author	Garavand, Ali and Aslani, Nasim and Nadri, Hamed and Abedini, Saeideh and Dehghan, Shirin
doi	10.1016/J.IMU.2022.100943
file	:C\:/Users/Unibague/AppData/Local/Mendeley Ltd./Mendeley Desktop/Downloaded/Garavand et al. - 2022 - Acceptance of telemedicine technology among physicians A systematic review.pdf:pdf
issn	23529148
journal	Informatics in Medicine Unlocked
keywords	Physician,Technology acceptance models,Telehealth,Telemedicine
month	jan
publisher	Elsevier Ltd
title	Acceptance of telemedicine technology among physicians: A systematic review
volume	30
year	2022
**article**	**Sun2020**
abstract	Three-dimensional (3D) printing has been increasingly used in medicine with applications in many different fields ranging from orthopaedics and tumours to cardiovascular disease. Realistic 3D models can be printed with different materials to replicate anatomical structures and pathologies with high accuracy. 3D printed models generated from medical imaging data acquired with computed tomography, magnetic resonance imaging or ultrasound augment the understanding of complex anatomy and pathology, assist preoperative planning and simulate surgical or interventional procedures to achieve precision medicine for improvement of treatment outcomes, train young or junior doctors to gain their confidence in patient management and provide medical education to medical students or healthcare professionals as an effective training tool. This article provides an overview of patient-specific 3D printed models with a focus on the applications in cardiovascular disease including: 3D printed models in congeni-

	tal heart disease, coronary artery disease, pulmonary embolism, aortic aneurysm and aortic dissection, and aortic valvular disease. Clinical value of the patient-specific 3D printed models in these areas is presented based on the current literature, while limitations and future research in 3D printing including bioprinting of cardiovascular disease are highlighted.
author	Sun, Zhonghua
doi	10.3390/biom10111577
issn	2218273X
journal	Biomolecules
number	11
pages	1--34
pmid	33233652
title	Clinical applications of patient-specific 3d printed models in cardiovascular disease: Current status and future directions
volume	10
year	2020
**article**	**Tamura2023**
author	Tamura, Yuichi and Nomura, Akihiro and Kagiyama, Nobuyuki and Mizuno, Atsushi and Node, Koichi
doi	10.1016/j.jjcc.2023.12.002
issn	09145087
journal	Journal of Cardiology
number	xxxx
pmid	38135148
publisher	The Authors
title	Digitalomics, digital intervention, and designing future: The next frontier in cardiology
year	2023
**article**	**Sandeep2024**
abstract	In routine clinical practice, the diagnosis and treatment of cardiovascular disease (CVD) rely on data in a variety of formats. These formats com-

	prise invasive angiography, laboratory data, non-invasive imaging diagnostics, and patient history. Artificial intelligence (AI) is a field of computer science that aims to mimic human thought processes, learning capacity, and knowledge storage. In cardiovascular medicine, artificial intelligence (AI) algorithms have been used to discover novel genotypes and phenotypes in established diseases enhance patient care, enable cost effectiveness, and lower readmission and mortality rates. AI will lead to a paradigm change toward precision cardiovascular medicine in the near future. The promise application of AI in cardiovascular medicine is immense; however, failure to recognize and ignorance of the challenges may overshadow its potential clinical impact. AI can facilitate every stage in cardiology in the imaging process, from acquisition and reconstruction, to segmentation, measurement, interpretation, and subsequent clinical pathways. Along with new possibilities, new threats arise, acknowledging and understanding them is as important as understanding the machine learning (ML) methodology itself. Therefore, attention is also paid to the current opinions and guidelines regarding the validation and safety of AI. This paper provides a outline for clinicians on relevant aspects of AI and machine learning, selection of applications and methods in cardiology to date, and identifies how cardiovascular medicine could incorporate AI in the future. With progress continuing in this emerging technology, the impact for cardiovascular medicine is highlighted to provide insight for the practicing clinician and to identify potential patient benefits.
author	Sandeep, Bhushan and Liu, Xian and Huang, Xin and Wang, Xiaowei and Mao, Long and Xiao, Zongwei
doi	10.1016/j.cpcardiol.2023.102349
issn	15356280
journal	Current Problems in Cardiology
number	2
pages	102349
pmid	38103818
publisher	Elsevier Inc.
title	Feasibility of artificial intelligence its current status, clinical applications, and future direction in cardiovascular disease
volume	49
year	2024
**article**	**Gorshkov2019**
abstract	This article proposes new solutions for visualization of biomedical signals in development and operation of cardiodiagnostics telemedicine systems. Visual evaluation of bioparameters is based on multilevel wavelet analysis of recorded patient's acoustic signals describing current state of the cardiovascular system, respiratory system and speech system. Proposed visualization solutions are implemented in form of ACUSTOCARD and ACUSTOMED telemedicine systems. Developed ACUSTOCARD telemedicine system being a set of software and hardware tools helps to

	find any cardiovascular diseases at earlier stages. ACUSTOMED is a further step of ACUSTOCARD telemedicine system, which additionally allows receiving acoustic sonograms of breath sounds and wavelet- sonograms of speech signals to evaluate the level of patient's emotional tension. Scope of application of ACUSTOMED telemedicine system includes as follows: instant diagnostics of the cardiovascular system; operational monitoring of the heart state for patients after the cardiac surgery; pediatric cardiology; sports medicine; home telemedicine. Experimental testing of biomedical signal visualization technology in cardiodiagnostics telemedicine systems confirms its high efficiency.
author	Gorshkov, Y. G.
doi	10.26583/sv.11.2.05
issn	20793537
journal	Scientific Visualization
number	2
pages	56--72
title	New solutions for visualization of biomedical signals in telemedicine systems
volume	11
year	2019
**article**	**AlKnawy2022**
abstract	IMPORTANCE COVID-19 has highlighted widespread chronic underinvestment in digital health that hampered public health responses to the pandemic. Recognizing this, the Riyadh Declaration on Digital Health, formulated by an international interdisciplinary team of medical, academic, and industry experts at the Riyadh Global Digital Health Summit in August 2020, provided a set of digital health recommendations for the global health community to address the challenges of current and future pandemics. However, guidance is needed on how to implement these recommendations in practice. OBJECTIVE To develop guidance for stakeholders on how best to deploy digital health and data and support public health in an integrated manner to overcome the COVID-19 pandemic and future pandemics. EVIDENCE REVIEW Themes were determined by first reviewing the literature and Riyadh Global Digital Health Summit conference proceedings, with experts independently contributing ideas. Then, 2 rounds of review were conducted until all experts agreed on the themes and main issues arising using a nominal group technique to reach consensus. Prioritization was based on how useful the consensus recommendation might be to a policy maker. FINDINGS A diverse stakeholder group of 13 leaders in the fields of public health, digital health, and health care were engaged to reach a consensus on how to implement digital health recommendations to address the challenges of current and future pandemics. Participants reached a consensus on high-priority issues identified within 5 themes: team, transparency and trust, technology, techquity (the strategic development and deployment of technology in health care and health to achieve health equity), and transformation. Each theme contains concrete points of consensus to guide the local, national, and international adoption of digital health to address challenges of current and future pandemics. CONCLUSIONS AND RELEVANCE The consensus points described

	for these themes provide a roadmap for the implementation of digital health policy by all stakeholders, including governments. Implementation of these recommendations could have a significant impact by reducing fatalities and uniting countries on current and future battles against pandemics. Question What digital health recommendations should be adopted by the global health community to address the challenges of current and future pandemics? Findings By engaging a diverse stakeholder group of 13 leaders in the fields of public health, digital health, and health care, a consensus was reached on how to implement digital health recommendations to address the challenges of current and future pandemics across 5 main themes: team, transparency and trust, technology, techquity (the strategic development and deployment of technology in health care and health to achieve health equity), and transformation. Meaning This consensus statement provides a roadmap for the implementation of digital health policy by stakeholders, including governments, to prepare for and address current and future pandemics.
author	Al Knawy}, Bandar and Mollie, ; and Mckillop, Marian and Abduljawad, Joud and Tarkoma, Sasu and Adil, Mahmood and Schaper, Louise and Chee, Adam and Bates, David W and Klag, Michael and Lee, Uichin and Kozlakidis, Zisis and Crooks, George and Rhee, Kyu
doi	10.1001/jamanetworkopen.2022.0214
journal	jamanetwork.com
number	2
pages	220214
title	Successfully implementing digital health to ensure future global health security during pandemics: a consensus statement
url	https://jamanetwork.com/journals/jamanetworkopen/article-abstract/2789277
volume	5
year	2022
**article**	**Medvedev2022**
abstract	Citation: Li, X.; Liu, X.; Deng, X.; Fan, Y. Interplay between Artificial Intelligence and Biomechanics Modeling in the Cardiovascular Disease Prediction. Biomedicines 2022, 10, 2157. https://doi. Abstract: Cardiovascular disease (CVD) is the most common cause of morbidity and mortality worldwide, and early accurate diagnosis is the key point for improving and optimizing the prognosis of CVD. Recent progress in artificial intelligence (AI), especially machine learning (ML) technology, makes it possible to predict CVD. In this review, we first briefly introduced the overview development of artificial intelligence. Then we summarized some ML applications in cardiovascular diseases, including ML–based models to directly predict CVD based on risk factors or medical imaging findings and the ML–based hemodynamics with vascular geometries, equations, and methods for indirect assessment of CVD. We also discussed case studies where ML could be used as the surrogate for computational fluid dynamics in data–driven models and physics–driven models. ML models could be a surrogate for computational fluid dynamics, accelerate the process of disease prediction, and reduce manual intervention. Lastly, we briefly summarized the research difficulties and prospected the future development of AI technology in cardiovascular diseases.

author	Medvedev, Nikolaevich and Zavalishina, Svetlana Yurievna and {Viktorovna Vorobieva}, Nadezhda and Li, Xiaoyin and Liu, Xiao and Deng, Xiaoyan and Fan, Yubo
doi	10.3390/biomedicines10092157
file	:C\:/Users/Unibague/AppData/Local/Mendeley Ltd./Mendeley Desktop/Downloaded/Medvedev et al. - 2022 - Interplay between artificial intelligence and biomechanics modeling in the cardiovascular disease prediction.pdf:pdf
journal	mdpi.comX Li, X Liu, X Deng, Y FanBiomedicines, 2022•mdpi.com
keywords	artificial intelligence,cardiovascular biomechanics modeling,cardiovascular diseases,machine learning
month	sep
number	9
publisher	MDPI
title	Interplay between artificial intelligence and biomechanics modeling in the cardiovascular disease prediction
url	https://www.mdpi.com/2227-9059/10/9/2157
volume	10
year	2022
**article**	**Tang2019**
abstract	At present, deep learning has been widely adopted in medical image processing. However, the current deep neural networks depend on a large number of labeled training data, but medical images segmentation tasks often suffer from the problem of small quantity of labeled data because labeling medical images is a very expensive and time-consuming task. In order to overcome this difficulty, this paper proposes a new image augmentation strategy based on statistical shape model and three-dimensional thin plate spline, which can generate many simulated images from a small number of real images. Firstly, the shape information of the real labeled images is modeled with the statistical shape model, and a series of simulated shapes are generated by sampling from this model. Secondly, the simulated shapes are filled with texture using three-dimensional thin plate spline to generate the simulated images. Finally, the simulated images and the real images are used together for training deep neural networks. The proposed framework is a general data augmentation method that can be used in any anatomical structure segmentation tasks with any deep neural network architecture. We used two different datasets, including prostate MRI dataset and liver CT dataset, and used two different deep network structures, including multi-scale 3D Convolutional Neural Networks (multi-scale 3D CNN) and U-net. The experimental results showed that the proposed data augmentation strategy can improve the accuracy of existing segmentation algorithms based on deep neural networks.
author	Tang, Zhixian and Chen, Kun and Pan, Mingyuan and Wang, Manning and Song, Zhijian

doi	10.1109/ACCESS.2019.2941154
issn	21693536
journal	IEEE Access
pages	133111--133121
publisher	IEEE
title	An Augmentation Strategy for Medical Image Processing Based on Statistical Shape Model and 3D Thin Plate Spline for Deep Learning
volume	7
year	2019
**article**	**Mohsen2023**
abstract	Citation: Mohsen, F.; Al-Saadi, B.; Abdi, N.; Khan, S.; Shah, Z. Artificial Intelligence-Based Methods for Precision Cardiovascular Medicine. J. Pers. Med. 2023, 13, 1268. https:// Abstract: Precision medicine has the potential to revolutionize the way cardiovascular diseases are diagnosed, predicted, and treated by tailoring treatment strategies to the individual characteristics of each patient. Artificial intelligence (AI) has recently emerged as a promising tool for improving the accuracy and efficiency of precision cardiovascular medicine. In this scoping review, we aimed to identify and summarize the current state of the literature on the use of AI in precision cardiovascular medicine. A comprehensive search of electronic databases, including Scopes, Google Scholar, and PubMed, was conducted to identify relevant studies. After applying inclusion and exclusion criteria, a total of 28 studies were included in the review. We found that AI is being increasingly applied in various areas of cardiovascular medicine, including the diagnosis, prognosis of cardiovascular diseases, risk prediction and stratification, and treatment planning. As a result, most of these studies focused on prediction (50%), followed by diagnosis (21%), phenotyping (14%), and risk stratification (14%). A variety of machine learning models were utilized in these studies, with logistic regression being the most used (36%), followed by random forest (32%), support vector machine (25%), and deep learning models such as neural networks (18%). Other models, such as hierarchical clustering (11%), Cox regression (11%), and natural language processing (4%), were also utilized. The data sources used in these studies included electronic health records (79%), imaging data (43%), and omics data (4%). We found that AI is being increasingly applied in various areas of cardiovascular medicine, including the diagnosis, prognosis of cardiovascular diseases, risk prediction and stratification, and treatment planning. The results of the review showed that AI has the potential to improve the performance of cardiovascular disease diagnosis and prognosis, as well as to identify individuals at high risk of developing cardiovascular diseases. However, further research is needed to fully evaluate the clinical utility and effectiveness of AI-based approaches in precision cardiovascular medicine. Overall, our review provided a comprehensive overview of the current state of knowledge in the field of AI-based methods for precision cardiovascular medicine and offered new insights for researchers interested in this research area.
author	Mohsen, F and Al-Saadi, B and Abdi, N and …, S Khan - Journal of Personalized and undefined 2023
doi	10.3390/jpm13081268

file	:C\:/Users/Unibague/AppData/Local/Mendeley Ltd./Mendeley Desktop/Downloaded/Mohsen et al. - 2023 - Artificial Intelligence-Based Methods for Precision Cardiovascular Medicine.pdf:pdf
journal	mdpi.comF Mohsen, B Al-Saadi, N Abdi, S Khan, Z ShahJournal of Personalized Medicine, 2023•mdpi.com
keywords	artificial intelligence,cardiovascular diseases,machine learning,precision medicine
month	aug
number	8
publisher	Multidisciplinary Digital Publishing Institute (MDPI)
title	Artificial Intelligence-Based Methods for Precision Cardiovascular Medicine
url	https://www.mdpi.com/2075-4426/13/8/1268
volume	13
year	2023
**article**	**Briganti2020a**
abstract	Artificial intelligence-powered medical technologies are rapidly evolving into applicable solutions for clinical practice. Deep learning algorithms can deal with increasing amounts of data provided by wearables, smartphones, and other mobile monitoring sensors in different areas of medicine. Currently, only very specific settings in clinical practice benefit from the application of artificial intelligence, such as the detection of atrial fibrillation, epilepsy seizures, and hypoglycemia, or the diagnosis of disease based on histopathological examination or medical imaging. The implementation of augmented medicine is long-awaited by patients because it allows for a greater autonomy and a more personalized treatment, however, it is met with resistance from physicians which were not prepared for such an evolution of clinical practice. This phenomenon also creates the need to validate these modern tools with traditional clinical trials, debate the educational upgrade of the medical curriculum in light of digital medicine as well as ethical consideration of the ongoing connected monitoring. The aim of this paper is to discuss recent scientific literature and provide a perspective on the benefits, future opportunities and risks of established artificial intelligence applications in clinical practice on physicians, healthcare institutions, medical education, and bioethics.
author	Briganti, Giovanni and {Le Moine}, Olivier
doi	10.3389/FMED.2020.00027/FULL
issn	2296858X
journal	Frontiers in Medicine
keywords	artificial intelligence,digital medicine,medical technologies,mobile health,monitoring

month	feb
publisher	Frontiers Media S.A.
title	Artificial Intelligence in Medicine: Today and Tomorrow
volume	7
year	2020
**article**	**Aria2017**
abstract	The use of bibliometrics is gradually extending to all disciplines. It is particularly suitable for science mapping at a time when the emphasis on empirical contributions is producing voluminous, fragmented, and controversial research streams. Science mapping is complex and unwieldly because it is multi-step and frequently requires numerous and diverse software tools, which are not all necessarily freeware. Although automated workflows that integrate these software tools into an organized data flow are emerging, in this paper we propose a unique open-source tool, designed by the authors, called bibliometrix, for performing comprehensive science mapping analysis. bibliometrix supports a recommended workflow to perform bibliometric analyses. As it is programmed in R, the proposed tool is flexible and can be rapidly upgraded and integrated with other statistical R-packages. It is therefore useful in a constantly changing science such as bibliometrics.
author	Aria, Massimo and Cuccurullo, Corrado
doi	10.1016/j.joi.2017.08.007
issn	18755879
journal	Journal of Informetrics
number	4
pages	959--975
publisher	Elsevier Ltd
title	bibliometrix: An R-tool for comprehensive science mapping analysis
volume	11
year	2017
**article**	**Garavand2022**
abstract	Background: Telemedicine is vital technology to deliver health services at a distance by health professionals, especially physicians, who are key players in Community health. Given the important role of telemedicine in improving health care, especially in the COVID-19 epidemic, an exami-

	nation of behavioral barriers and not using this technology among physicians can be important. Objectives: The aim of our systematic review is to identify the behavioral factors influencing the acceptance of telemedicine technology among physicians in different contexts. Methods: A literature search was conducted according to the PRISMA guidelines. The search was conducted without any time limitations up to the Dec of 2020 in Web of Science, PubMed, Scopus, and Embase scientific databases; by applying keywords. The article selection was made based on inclusion (telemedicine among physicians, using the acceptance behavioral theories), and exclusion (physicians not the end-users of technology, it is not about acceptance of technology) criteria by two authors independently. Data was gathered using a data extraction form, and the results were reported in tables and figures based on the study objectives. Results: From all the retrieved studies, 37 articles were included based on the inclusion and exclusion criteria. The United States and Spain have the most conducted studies about the acceptance of telemedicine from the physicians' point of view. The study results showed that the Technology Acceptance Model (TAM) and extended TAM model have the highest frequency. The main factors affecting the acceptance and use of telemedicine were perceived usefulness, attitude to use, compatibility, perceived ease of use, self-efficacy, subjective norms, perceived behavioral control, and facilitating condition. Conclusions: Identifying the most important factors that affect the acceptance of telemedicine from physicians' perspectives, as a key player in telemedicine projects, can help managers and policymakers make the right decisions about implementation of telemedicine successfully, especially in the initial phases. Future studies can also evaluate the aggregation of factors identified in this paper.
author	Garavand, Ali and Aslani, Nasim and Nadri, Hamed and Abedini, Saeideh and Dehghan, Shirin
doi	10.1016/j.imu.2022.100943
issn	23529148
journal	Informatics in Medicine Unlocked
number	January
pages	100943
publisher	Elsevier Ltd
title	Acceptance of telemedicine technology among physicians: A systematic review
volume	30
year	2022
**article**	**Yeung2022**
abstract	Background: Digital technology uses in cardiology have become a popular research focus in recent years. However, there has been no published bibliometric report that analyzed the corresponding academic literature in order to derive key publishing trends and characteristics of this scientific area. Objective: We used a bibliometric approach to identify and analyze the academic literature on digital technology uses in cardiology, and to unveil popular research topics, key authors, institutions, countries, and journals. We further captured the cardiovascular conditions and diagnostic

	tools most commonly investigated within this field. Methods: The Web of Science electronic database was queried to identify relevant papers on digital technology uses in cardiology. Publication and citation data were acquired directly from the database. Complete bibliographic data were exported to VOSviewer, a dedicated bibliometric software package, and related to the semantic content of titles, abstracts, and keywords. A term map was constructed for findings visualization. Results: The analysis was based on data from 12,529 papers. Of the top 5 most productive institutions, 4 were based in the United States. The United States was the most productive country (4224/12,529, 33.7%), followed by United Kingdom (1136/12,529, 9.1%), Germany (1067/12,529, 8.5%), China (682/12,529, 5.4%), and Italy (622/12,529, 5.0%). Cardiovascular diseases that had been frequently investigated included hypertension (152/12,529, 1.2%), atrial fibrillation (122/12,529, 1.0%), atherosclerosis (116/12,529, 0.9%), heart failure (106/12,529, 0.8%), and arterial stiffness (80/12,529, 0.6%). Recurring modalities were electrocardiography (170/12,529, 1.4%), angiography (127/12,529, 1.0%), echocardiography (127/12,529, 1.0%), digital subtraction angiography (111/12,529, 0.9%), and photoplethysmography (80/12,529, 0.6%). For a literature subset on smartphone apps and wearable devices, the Journal of Medical Internet Research (20/632, 3.2%) and other JMIR portfolio journals (51/632, 8.0%) were the major publishing venues. Conclusions: Digital technology uses in cardiology target physicians, patients, and the general public. Their functions range from assisting diagnosis, recording cardiovascular parameters, and patient education, to teaching laypersons about cardiopulmonary resuscitation. This field already has had a great impact in health care, and we anticipate continued growth.
author	Yeung, Andy Wai Kan and Kulnik, Stefan Tino and Parvanov, Emil D. and Fassl, Anna and Eibensteiner, Fabian and Völkl-Kernstock, Sabine and Kletecka-Pulker, Maria and Crutzen, Rik and Gutenberg, Johanna and Höppchen, Isabel and Niebauer, Josef and Smeddinck, Jan David and Willschke, Harald and Atanasov, Atanas G.
doi	10.2196/36086
issn	14388871
journal	Journal of Medical Internet Research
number	5
pmid	35544307
title	Research on Digital Technology Use in Cardiology: Bibliometric Analysis
volume	24
year	2022
**article**	**Leng2015**
abstract	Most heart diseases are associated with and reflected by the sounds that the heart produces. Heart auscultation, defined as listening to the heart sound, has been a very important method for the early diagnosis of cardiac dysfunction. Traditional auscultation requires substantial clinical experience and good listening skills. The emergence of the electronic stethoscope has paved the way for a new field of computer-aided auscultation.

	This article provides an in-depth study of (1) the electronic stethoscope technology, and (2) the methodology for diagnosis of cardiac disorders based on computer-aided auscultation. The paper is based on a comprehensive review of (1) literature articles, (2) market (state-of-the-art) products, and (3) smartphone stethoscope apps. It covers in depth every key component of the computer-aided system with electronic stethoscope, from sensor design, front-end circuitry, denoising algorithm, heart sound segmentation, to the final machine learning techniques. Our intent is to provide an informative and illustrative presentation of the electronic stethoscope, which is valuable and beneficial to academics, researchers and engineers in the technical field, as well as to medical professionals to facilitate its use clinically. The paper provides the technological and medical basis for the development and commercialization of a real-time integrated heart sound detection, acquisition and quantification system.
author	Leng, Shuang and Tan, Ru San and Chai, Kevin Tshun Chuan and Wang, Chao and Ghista, Dhanjoo and Zhong, Liang
doi	10.1186/s12938-015-0056-y
issn	1475925X
journal	BioMedical Engineering Online
number	1
pages	1--37
pmid	26159433
publisher	BioMed Central
title	The electronic stethoscope
volume	14
year	2015
**article**	**Leng2015a**
abstract	Most heart diseases are associated with and reflected by the sounds that the heart produces. Heart auscultation, defined as listening to the heart sound, has been a very important method for the early diagnosis of cardiac dysfunction. Traditional auscultation requires substantial clinical experience and good listening skills. The emergence of the electronic stethoscope has paved the way for a new field of computer-aided auscultation. This article provides an in-depth study of (1) the electronic stethoscope technology, and (2) the methodology for diagnosis of cardiac disorders based on computer-aided auscultation. The paper is based on a comprehensive review of (1) literature articles, (2) market (state-of-the-art) products, and (3) smartphone stethoscope apps. It covers in depth every key component of the computer-aided system with electronic stethoscope, from sensor design, front-end circuitry, denoising algorithm, heart sound segmentation, to the final machine learning techniques. Our intent is to provide an informative and illustrative presentation of the electronic stethoscope, which is valuable and beneficial to academics, researchers and engineers in the technical field, as well as to medical professionals to facilitate its use clinically. The paper provides the technological and medical basis for

	the development and commercialization of a real-time integrated heart sound detection, acquisition and quantification system.
author	Leng, Shuang and Tan, Ru San and Chai, Kevin Tshun Chuan and Wang, Chao and Ghista, Dhanjoo and Zhong, Liang
doi	10.1186/S12938-015-0056-Y
file	:C\:/Users/Unibague/AppData/Local/Mendeley Ltd./Mendeley Desktop/Downloaded/Leng et al. - 2015 - The electronic stethoscope.pdf:pdf
issn	1475925X
journal	BioMedical Engineering Online
keywords	Acoustic technique,Automatic system,Diagnosis,Heart auscultation,Heart disorder,Heart sound,Smartphone stethoscope apps
month	jul
number	1
pmid	26159433
publisher	BioMed Central Ltd.
title	The electronic stethoscope
volume	14
year	2015
**article**	**Gerc2020**
author	Gerc, V and Masic, I and Salihefendic, N and Socio-medica, M Zildzic - Materia and 2020, Undefined
journal	Materia socio-medica
title	Cardiovascular diseases (CVDs) in COVID-19 pandemic era
url	https://www.ncbi.nlm.nih.gov/pmc/articles/PMC7428924/
year	2020
**article**	**Azmi2022a**
abstract	There is a considerable rise in cardiovascular diseases in the world. It is pertinently essential to make cardiovascular prediction accurate to the maximum. A forecast based on machine learning techniques can be beneficial in detecting cardiovascular disease (CVD) with maximum precision and accuracy. The disease's effective prediction helps in early diagnosis, which cuts down the mortality rate. A health history and the causes of heart disease require the efficient detection and prediction of CVD. Data analytics is beneficial for making predictions based on a massive amount

	of data, and it aids health clinics in disease prognosis. Regularly, a large volume of patient-related data is maintained. The information gathered can be used to forecast the emergence of upcoming diseases. Our study presents a detailed comparative study of Cardiovascular Disease by comparing the various machine learning techniques mainly comprising of classification and predictive algorithms. The study shows an in-depth analysis of around forty-one papers related to cardiovascular disease by using machine learning techniques. This study evaluates the selected publications rigorously and identifies gaps in the available literature, making it useful for researchers to develop and apply in clinical fields, primarily on datasets related to heart disease. The current study will aid medical practitioners in predicting heart threats ahead of time, allowing them to take preventative measures.
author	Azmi, Javed and Arif, Muhammad and Nafis, Md Tabrez and Alam, M. Afshar and Tanweer, Safdar and Wang, Guojun
doi	10.1016/J.MEDENGPHY.2022.103825
issn	18734030
journal	Medical Engineering and Physics
keywords	CVD,Cardiovascular disease,Classification,Disease prediction,Machine learning
month	jul
pmid	35781385
publisher	Elsevier Ltd
title	A systematic review on machine learning approaches for cardiovascular disease prediction using medical big data
volume	105
year	2022
**article**	**Wong2020a**
abstract	Artificial intelligence (AI) is becoming a vital concept in medicine leading to a rapid emergence of important tools for medical diagnostics. Now, as a crucial machine learning tool in the field of computer vision, deep learning (DL) is being widely used in medical imaging. Furthermore, as reported in the medical literature, DL has been widely used in medical related research. However, the practical application of DL in clinical diagnosis is relatively small, and it is a new field that may have some challenges. How to effectively perform medical image analysis is a major problem in the field of disease diagnosis, and further diagnostic methods need to be developed. At this stage, DL could be viewed as a black box requiring knowledge of its internal workings, and hence presents some crucial technical challenges that need further methodological development. Thereafter with proper diagnostics, pre-operative computerized simulation planning can be carried out for use of appropriate surgical intervention technology. This paper presents important questions on cardiovascular disease (CVD) diagnostics, using this powerful and yet not adequately understood technology. It discusses issues brought by the paradigm shift of AI vis-à-vis DL in CVD diagnostics, provides possible solutions to potential issues,

	and envisions the future of the related machine intelligence applications. The discussed problems are dissected into the modular aspects of DL in relation to CVD image classification, segmentation, and detection. A proper perspective on management of these issues is the key to a successful technological implementation of DL in modern medical science.
author	Wong, Kelvin K.L. and Fortino, Giancarlo and Abbott, Derek
doi	10.1016/J.FUTURE.2019.09.047
issn	0167739X
journal	Future Generation Computer Systems
keywords	Artificial intelligence,Big data in medicine,Cardiovascular diagnosis,Deep learning,Image analysis
month	sep
pages	802--811
publisher	Elsevier B.V.
title	Deep learning-based cardiovascular image diagnosis: A promising challenge
volume	110
year	2020
**article**	**Morcillo2020**
author	Morcillo, César and González, José Luis
doi	10.1016/j.medcli.2019.07.004
issn	15788989
journal	Medicina Clinica
number	7
pages	257--259
pmid	31488262
publisher	Elsevier España, S.L.U.
title	New digital healthcare technologies
volume	154

year	2020
**article**	**Lindquist2021b**
abstract	With the rate of cardiovascular diseases in the U.S increasing throughout the years, there is a need for developing more advanced treatment plans that can be tailored to specific patients and scenarios. The development of 3D printing is rapidly gaining acceptance into clinical cardiology. In this review, key technologies used in 3D printing are briefly summarized, particularly, the use of artificial intelligence (AI), open-source tools like MeshLab and MeshMixer, and 3D printing techniques such as fused deposition molding (FDM) and polyjet are reviewed. The combination of 3D printing, multiple image integration, and augmented reality may greatly enhance data visualization during diagnosis, treatment planning, and surgical procedures for cardiology.
author	Lindquist, Ellen M. and Gosnell, Jordan M. and Khan, Sana K. and Byl, John L. and Zhou, Weihua and Jiang, Jingfeng and Vettukattil, Joseph J.
doi	10.1016/J.STLM.2021.100034
file	:C\:/Users/Unibague/AppData/Local/Mendeley Ltd./Mendeley Desktop/Downloaded/Lindquist et al. - 2021 - 3D printing in cardiology A review of applications and roles for advanced cardiac imaging.pdf:pdf
issn	26669641
journal	Annals of 3D Printed Medicine
keywords	3D printing,AI,Augmented reality,Cardiovascular disease,Diagnosis
month	dec
publisher	Elsevier Inc.
title	3D printing in cardiology: A review of applications and roles for advanced cardiac imaging
volume	4
year	2021
**article**	**MohsenFarida;.Al-SaadiBalqees;AbdiNima;.Khan2023**
author	Mohsen, Farida;.Al-Saadi, Balqees; Abdi, Nima;. Khan}, Sulaimán;. Shah Zubair;.
doi	https://doi.org/10.3390/jpm13081268
journal	Personalized Medicine
title	Métodos basados en inteligencia artificial para la medicina cardiovascular de precisión.pdf
volume	13

year	2023
**article**	**Sandeep2024a**
abstract	In routine clinical practice, the diagnosis and treatment of cardiovascular disease (CVD) rely on data in a variety of formats. These formats comprise invasive angiography, laboratory data, non-invasive imaging diagnostics, and patient history. Artificial intelligence (AI) is a field of computer science that aims to mimic human thought processes, learning capacity, and knowledge storage. In cardiovascular medicine, artificial intelligence (AI) algorithms have been used to discover novel genotypes and phenotypes in established diseases enhance patient care, enable cost effectiveness, and lower readmission and mortality rates. AI will lead to a paradigm change toward precision cardiovascular medicine in the near future. The promise application of AI in cardiovascular medicine is immense; however, failure to recognize and ignorance of the challenges may overshadow its potential clinical impact. AI can facilitate every stage in cardiology in the imaging process, from acquisition and reconstruction, to segmentation, measurement, interpretation, and subsequent clinical pathways. Along with new possibilities, new threats arise, acknowledging and understanding them is as important as understanding the machine learning (ML) methodology itself. Therefore, attention is also paid to the current opinions and guidelines regarding the validation and safety of AI. This paper provides a outline for clinicians on relevant aspects of AI and machine learning, selection of applications and methods in cardiology to date, and identifies how cardiovascular medicine could incorporate AI in the future. With progress continuing in this emerging technology, the impact for cardiovascular medicine is highlighted to provide insight for the practicing clinician and to identify potential patient benefits.
author	Sandeep, Bhushan and Liu, Xian and Huang, Xin and Wang, Xiaowei and Mao, Long and Xiao, Zongwei
doi	https://doi.org/10.1016/j.cpcardiol.2023.102349
issn	0146-2806
journal	Current Problems in Cardiology
keywords	Artificial intelligence,Cardiovascular imaging,Heart failure,Machine learning
number	2
pages	102349
title	Feasibility of artificial intelligence its current status, clinical applications, and future direction in cardiovascular disease
url	https://www.sciencedirect.com/science/article/pii/S0146280623007661
volume	49
year	2024
**article**	**Briganti**

author	Briganti, G and in Medicine, O Le Moine - Frontiers and undefined 2020
journal	frontiersin.orgG Briganti, O Le MoineFrontiers in medicine, 2020•frontiersin.org
title	Artificial intelligence in medicine: today and tomorrow
url	https://www.frontiersin.org/articles/10.3389/fmed.2020.00027/full?trk=article-ssr-frontend-pulse_x-social-details_comments-action_comment-text
**article**	**GomezGarcia2021**
abstract	This article presents the hardware-software design and implementation of an open, integrated, and scalable healthcare platform oriented to multiple point-care scenarios for healthcare promotion and cardiovascular disease prevention. The platform has the capability to provide continuous monitoring, extended device integration, strategies based on artificial intelligence for the information analysis and cybersecurity support, delivering a secure end-to-end hardware-software solution. This platform is used to perform the remote patient health monitoring and supervision by doctors, triage procedures in hospitals, or self-care monitoring using personal devices such as tablets and cellphones. The proposed hardware architecture facilitates the integration of biomedical data acquired from different health-point cares, collecting relevant information for the detection of cardiovascular risk through deep-learning algorithms. All these characteristics make our development a strong tool to perform epidemiological profiling and future implementation of strategies for comprehensive cardiovascular risk intervention. The components of the platform are described, and their main functionalities are highlighted.
author	Gomez Garcia}, Carlos A. and {Askar Rodriguez}, Miguel and {Velasco Medina}, Jaime
doi	10.1109/JBHI.2021.3051967
issn	21682208
journal	IEEE Journal of Biomedical and Health Informatics
number	7
pages	2758--2767
pmid	33449888
title	Platform for Healthcare Promotion and Cardiovascular Disease Prevention
volume	25
year	2021
**article**	**Moher2009a**
author	Moher, David and Liberati, Alessandro and Tetzlaff, Jennifer and Altman, Douglas G. and Antes, Gerd and Atkins, David and Barbour, Virginia and Barrowman, Nick and Berlin, Jesse A. and Clark, Jocalyn and Clarke, Mike and Cook, Deborah and D'Amico, Roberto and Deeks, Jonathan J.

	and Devereaux, P. J. and Dickersin, Kay and Egger, Matthias and Ernst, Edzard and Gøtzsche, Peter C. and Grimshaw, Jeremy and Guyatt, Gordon and Higgins, Julian and Ioannidis, John P.A. and Kleijnen, Jos and Lang, Tom and Magrini, Nicola and McNamee, David and Moja, Lorenzo and Mulrow, Cynthia and Napoli, Maryann and Oxman, Andy and Pham, Bá and Rennie, Drummond and Sampson, Margaret and Schulz, Kenneth F. and Shekelle, Paul G. and Tovey, David and Tugwell, Peter
doi	10.1371/JOURNAL.PMED.1000097
file	:C\:/Users/Unibague/AppData/Local/Mendeley Ltd./Mendeley Desktop/Downloaded/Moher et al. - 2009 - Preferred reporting items for systematic reviews and meta-analyses The PRISMA statement.pdf:pdf
issn	15491676
journal	PLoS Medicine
month	jul
number	7
pmid	19621072
publisher	Public Library of Science
title	Preferred reporting items for systematic reviews and meta-analyses: The PRISMA statement
volume	6
year	2009
**article**	**Aria2017a**
abstract	The use of bibliometrics is gradually extending to all disciplines. It is particularly suitable for science mapping at a time when the emphasis on empirical contributions is producing voluminous, fragmented, and controversial research streams. Science mapping is complex and unwieldly because it is multi-step and frequently requires numerous and diverse software tools, which are not all necessarily freeware. Although automated workflows that integrate these software tools into an organized data flow are emerging, in this paper we propose a unique open-source tool, designed by the authors, called bibliometrix, for performing comprehensive science mapping analysis. bibliometrix supports a recommended workflow to perform bibliometric analyses. As it is programmed in R, the proposed tool is flexible and can be rapidly upgraded and integrated with other statistical R-packages. It is therefore useful in a constantly changing science such as bibliometrics.
author	Aria, Massimo and Cuccurullo, Corrado
doi	10.1016/J.JOI.2017.08.007
issn	18755879

journal	Journal of Informetrics
keywords	Bibliographic coupling,Bibliometrics,Co-citation,R package,Science mapping,Workflow
month	nov
number	4
pages	959--975
publisher	Elsevier Ltd
title	bibliometrix: An R-tool for comprehensive science mapping analysis
volume	11
year	2017
**article**	**Gomez-Garcia2021**
abstract	This article presents the hardware-software design and implementation of an open, integrated, and scalable healthcare platform oriented to multiple point-care scenarios for healthcare promotion and cardiovascular disease prevention. The platform has the capability to provide continuous monitoring, extended device integration, strategies based on artificial intelligence for the information analysis and cybersecurity support, delivering a secure end-to-end hardware-software solution. This platform is used to perform the remote patient health monitoring and supervision by doctors, triage procedures in hospitals, or self-care monitoring using personal devices such as tablets and cellphones. The proposed hardware architecture facilitates the integration of biomedical data acquired from different health-point cares, collecting relevant information for the detection of cardiovascular risk through deep-learning algorithms. All these characteristics make our development a strong tool to perform epidemiological profiling and future implementation of strategies for comprehensive cardiovascular risk intervention. The components of the platform are described, and their main functionalities are highlighted.
author	Gomez-Garcia, Carlos A. and Askar-Rodriguez, Miguel and Velasco-Medina, Jaime
doi	10.1109/JBHI.2021.3051967
issn	21682208
journal	IEEE Journal of Biomedical and Health Informatics
keywords	E-Health,Health kiosk,Healthcare systems,IoMT,M-Health
month	jul
number	7

pages	2758--2767
pmid	33449888
publisher	Institute of Electrical and Electronics Engineers Inc.
title	Platform for Healthcare Promotion and Cardiovascular Disease Prevention
volume	25
year	2021
**article**	**Javaid2022a**
abstract	Machine learning (ML) refers to computational algorithms that iteratively improve their ability to recognize patterns in data. The digitization of our healthcare infrastructure is generating an abundance of data from electronic health records, imaging, wearables, and sensors that can be analyzed by ML algorithms to generate personalized risk assessments and promote guideline-directed medical management. ML's strength in generating insights from complex medical data to guide clinical decisions must be balanced with the potential to adversely affect patient privacy, safety, health equity, and clinical interpretability. This review provides a primer on key advances in ML for cardiovascular disease prevention and how they may impact clinical practice.
author	Javaid, Aamir and Zghyer, Fawzi and Kim, Chang and Spaulding, Erin M. and Isakadze, Nino and Ding, Jie and Kargillis, Daniel and Gao, Yumin and Rahman, Faisal and Brown, Donald E. and Saria, Suchi and Martin, Seth S. and Kramer, Christopher M. and Blumenthal, Roger S. and Marvel, Francoise A.
doi	10.1016/J.AJPC.2022.100379
file	:C\:/Users/Unibague/AppData/Local/Mendeley Ltd./Mendeley Desktop/Downloaded/Javaid et al. - 2022 - Medicine 2032 The future of cardiovascular disease prevention with machine learning and digital health technology.pdf:pdf
issn	26666677
journal	American Journal of Preventive Cardiology
keywords	Artificial intelligence,Cardiology,Cardiovascular disease,Digital health,Machine learning,Prevention,Smartphones,Smartwatches,Wearables
month	dec
publisher	Elsevier B.V.
title	Medicine 2032: The future of cardiovascular disease prevention with machine learning and digital health technology
volume	12

year	2022
**article**	**MorcilloSerra2020**
author	Morcillo Serra}, César and {González Romero}, José Luis
doi	10.1016/J.MEDCLI.2019.07.004
issn	15788989
journal	Medicina Clinica
month	apr
number	7
pages	257--259
pmid	31488262
publisher	Ediciones Doyma, S.L.
title	New digital healthcare technologies
volume	154
year	2020
**book**	**WHO2021a**
author	WHO
isbn	9789240020924
title	Global strategy on digital health 2020-2025
year	2021
**article**	**Baladron2021a**
abstract	Technological progress in medicine is constantly garnering pace, requiring that physicians constantly update their knowledge. The new wave of technologies breaking through into clinical practice includes the following: a) mHealth, which allows constant monitoring of biological parameters, anytime, anyplace, of hundreds of patients at the same time; b) artificial intelligence, which, powered by new deep learning techniques, are starting to beat human experts at their own game: diagnosis by imaging or electrocardiography; c) 3-dimensional printing, which may lead to patient-specific prostheses; d) systems medicine, which has arisen from big data, and which will open the way to personalized medicine by bringing together genetic, epigenetic, environmental, clinical and social data into complex integral mathematical models to design highly personalized thera-

	pies. This state-of-the-art review aims to summarize in a single document the most recent and most important technological trends that are being applied to cardiology, and to provide an overall view that will allow readers to discern at a glance the direction of cardiology in the next few years.
author	Baladrón, Carlos and {Gómez de Diego}, José Juan and Amat-Santos, Ignacio J.
doi	10.1016/J.RECESP.2020.06.017
issn	15792242
journal	Revista Espanola de Cardiologia
keywords	Artificial intelligence,Big data,Systems medicine,mHealth
month	jan
number	1
pages	81--89
pmid	33008773
publisher	Ediciones Doyma, S.L.
title	Big data and new information technology: what cardiologists need to know
volume	74
year	2021
**article**	**Yin2019**
abstract	Background: Digital health is poised to transform health care and redefine personalized health. As Internet and mobile phone usage increases, as technology develops new ways to collect data, and as clinical guidelines change, all areas of medicine face new challenges and opportunities. Inflammatory bowel disease (IBD) is one of many chronic diseases that may benefit from these advances in digital health. This review intends to lay a foundation for clinicians and technologists to understand future directions and opportunities together. Objective: This review covers digital health apps that have been used in IBD, how they have fit into a clinical care framework, and the challenges that clinicians and technologists face in approaching future opportunities. Methods: We searched PubMed, Scopus, and ClinicalTrials.gov to identify digital health apps that have been studied and were published in the literature from January 1, 2010, to April 19, 2019. The search terms were "mobile health" OR "eHealth" OR "digital health" OR "smart phone" OR "mobile app" OR "mobile applications" OR "mHealth" OR "smartphones" AND "IBD" OR "Inflammatory bowel disease" OR "Crohn's Disease" (CD) OR "Ulcerative Colitis" (UC) OR "UC" OR "CD," followed by further analysis of citations from the results. We searched the Apple iTunes app store to identify a limited selection of commercial apps to include for discussion. Results: A total of 68 articles met the inclusion criteria. A total of 11 digital health apps were identified in the literature and 4 commercial apps were selected to be described in

	this review. While most apps have some educational component, the majority of apps focus on eliciting patient-reported outcomes related to disease activity, and a few are for treatment management. Significant benefits have been seen in trials relating to education, quality of life, quality of care, treatment adherence, and medication management. No studies have reported a negative impact on any of the above. There are mixed results in terms of effects on office visits and follow-up. Conclusions: While studies have shown that digital health can fit into, complement, and improve the standard clinical care of patients with IBD, there is a need for further validation and improvement, from both a clinical and patient perspective. Exploring new research methods, like microrandomized trials, may allow for more implementation of technology and rapid advancement of knowledge. New technologies that can o…
author	Yin, Andrew Lukas and Hachuel, David and Pollak, John P. and Scherl, Ellen J. and Estrin, Deborah
doi	10.2196/14630
issn	14388871
journal	Journal of Medical Internet Research
number	8
pages	1--19
pmid	31429410
title	Digital health apps in the clinical care of inflammatory bowel disease: Scoping review
volume	21
year	2019
**article**	**Tamura2024**
abstract	The discipline of cardiology stands at a transformative juncture, primarily influenced by the surge in digital health technologies. These innovations hold the promise to redefine the realms of cardiovascular research and patient care, ushering in an era of individualized and data-driven treatments. This review delves into the heart of this evolution, introducing a comprehensive design for the future of cardiology. Emphasizing the emerging domains of “digitalomics” and “digital intervention”, it explores how the integration of patient data, artificial intelligence-enabled diagnostics, and telehealth can lead to more streamlined and personalized cardiovascular health. The “digital-twin” model, a highlight of this approach, encapsulates individual patient characteristics, allowing for targeted treatments. The role of interdisciplinary collaboration in cardiovascular medicine is also underlined, emphasizing the importance of merging traditional cardiology with technological advancements. The convergence of traditional cardiology methods and digital health technologies, facilitated by a transdisciplinary approach, is set to chart a new course in cardiovascular health, emphasizing individualized care and improved clinical outcomes.
author	Tamura, Yuichi and Nomura, Akihiro and Kagiyama, Nobuyuki and Mizuno, Atsushi and Node, Koichi

doi	10.1016/J.JJCC.2023.12.002
file	:C\:/Users/Unibague/AppData/Local/Mendeley Ltd./Mendeley Desktop/Downloaded/Tamura et al. - 2024 - Digitalomics, digital intervention, and designing future The next frontier in cardiology.pdf:pdf
issn	18764738
journal	Journal of Cardiology
keywords	Artificial intelligence,Cardiovascular disease,Digital health,Digital intervention,Digitalomics
month	may
number	5
pages	318--322
pmid	38135148
publisher	Japanese College of Cardiology (Nippon-Sinzobyo-Gakkai)
title	Digitalomics, digital intervention, and designing future: The next frontier in cardiology
volume	83
year	2024
**article**	**Rubies-Prat2005**
author	Rubiés-Prat, J
doi	https://doi.org/10.1016/S0211-3449(05)73753-X
issn	0304-5412
journal	Medicine - Programa de Formación Médica Continuada Acreditado
number	38
pages	2506--2513
title	Factores de riesgo cardiovascular
url	https://www.sciencedirect.com/science/article/pii/S021134490573753X
volume	9

year	2005
**article**	**Turcu2023a**
abstract	Citation: Turcu, A.-M.; Ilie, A.C.; S , tef˘ aniu, R.; T , ˘ aranu, S.M.; Sandu, I.A.; Alexa-Stratulat, T.; Pîslaru, A.I.; Alexa, I.D. The Impact of Heart Rate Variability Monitoring on Preventing Severe Cardiovascular Events. Diagnostics 2023, 13, 2382. https:// Abstract: The increase in the incidence of cardiovascular diseases worldwide raises concerns about the urgent need to increase definite measures for the self-determination of different parameters, especially those defining cardiac function. Heart rate variability (HRV) is a non-invasive method used to evaluate autonomic nervous system modulation on the cardiac sinus node, thus describing the oscillations between consecutive electrocardiogram R-R intervals. These fluctuations are undetectable except when using specialized devices, with ECG Holter monitoring considered the gold standard. HRV is considered an independent biomarker for measuring cardiovascular risk and for screening the occurrence of both acute and chronic heart diseases. Also, it can be an important predictive factor of frailty or neurocognitive disorders, like anxiety and depression. An increased HRV is correlated with rest, exercise, and good recovery, while a decreased HRV is an effect of stress or illness. Until now, ECG Holter monitoring has been considered the gold standard for determining HRV, but the recent decade has led to an accelerated development of technology using numerous devices that were created specifically for the pre-hospital self-monitoring of health statuses. The new generation of devices is based on the use of photoplethysmography, which involves the determination of blood changes at the level of blood vessels. These devices provide additional information about heart rate (HR), blood pressure (BP), peripheral oxygen saturation (SpO 2), step counting, physical activity, and sleep monitoring. The most common devices that have this technique are smartwatches (used on a large scale) and chest strap monitors. Therefore, the use of technology and the self-monitoring of heart rate and heart rate variability can be an important first step in screening cardiovascular pathology and reducing the pressure on medical services in a hospital. The use of telemedicine can be an alternative, especially among elderly patients who are associated with walking disorders, frailty, or neurocognitive disorders.
author	Turcu, Ana-Maria and {Carmen Ilie}, Adina and Marie, Sabinne T and {Alexandra Sandu}, Ioana and Alexa-Stratulat, Teodora and {Iuliana Pîslaru}, Anca and {Dana Alexa}, Ioana
doi	10.3390/diagnostics13142382
file	:C\:/Users/Unibague/AppData/Local/Mendeley Ltd./Mendeley Desktop/Downloaded/Turcu et al. - 2023 - The impact of heart rate variability monitoring on preventing severe cardiovascular events(2).pdf:pdf
journal	mdpi.comAM Turcu, AC Ilie, R Ștefăniu, SM Țăranu, IA Sandu, T Alexa-Stratulat, AI Pîslaru, ID AlexaDiagnostics, 2023•mdpi.com
keywords	heart rate variability,novel wearable,wearable devices
month	jul
number	14
publisher	Multidisciplinary Digital Publishing Institute (MDPI)

title	The impact of heart rate variability monitoring on preventing severe cardiovascular events
url	https://www.mdpi.com/2075-4418/13/14/2382
volume	13
year	2023
**article**	**Dhingra2020**
abstract	Digital technology has been a revolutionary foray in education, industry, research and recently, healthcare. Digital health encompasses various aspects of technology like information and communication, mobile health, data-recording and telemedicine. There has been an exponential and unregulated increase in digital health services in last few years which have raised concerns over data privacy, ethical standards and quality of services. The World Health Organization recently released the global strategy on digital health as a visionary document that provides a framework for countries to implement and expand digital health services. The following update briefly highlights the salient features of the update.
author	Dhingra, Dhulika and Dabas, Aashima
doi	10.1007/s13312-020-1789-7
issn	09747559
journal	Indian Pediatrics
number	4
pages	356--358
pmid	32284477
title	Global Strategy on Digital Health
volume	57
year	2020
**misc**	**WHO2021**
author	WHO
title	Cardiovascular diseases (CVDs)
year	2021
**article**	**RabeloPadua2012**

abstract	RESUMEN RESUMEN RESUMEN RESUMEN Se realizó una extensa búsqueda bibliográfica sobre las enferme-dades no trasmisibles. Los datos científicos actuales indican que cuatro tipos de enfermedades no transmisibles (enfermedades cardiovascula-res, cánceres, enfermedades respiratorias crónicas y diabetes) son las principales causas de mortalidad En el año 2000, de las 3 537 000 muertes registradas en Latinoamérica y el Caribe, el 67 % fueron causadas por estos padecimientos crónicos. Las enfermedades crónicas no trasmisibles contribuyeron a la carga global de enfermedad con 76 % de los años de vida ajustados a la discapacidad; en sus fases graves tienen por causa factores de riesgos comunes y modificables. Palabras clave: enfermedades no transmisibles, enfermedades cróni-cas, años de vida ajustados a la discapacidad ABSTRACT ABSTRACT ABSTRACT ABSTRACT An extensive bibliographic search on noncommunicable diseases was carried out. Current evidence indicates that four types of noncommunicable diseases (cardiovascular diseases, cancers, chronic respiratory diseases and diabetes) are the main causes of death. In 2000, 67 % of the 3,537,000 deaths in Latin America and the Caribbean were caused by these chronic sufferings. Chronic noncommunicable diseases contributed to the global burden of disease with 76 % of the life years adjusted to disability; in severe stages they are caused by common and modifiable risk factors. Los rápidos cambios que amenazan a la salud mun-dial exigen una respuesta igualmente rápida que ante todo debe estar orientada al futuro. No es probable que las grandes epidemias de mañana se asemejen a las que hasta ahora han asolado al mundo gracias a los avances del control de las enfermedades infecciosas. Si bien el riesgo de brotes, por ejemplo, de una nueva pandemia de gripe exigirá una vigilancia constante, la epidemia 'invi-sible' de cardiopatías, accidentes cerebro vasculares, diabetes, cáncer y otras enfermedades crónicas, será la que en un futuro previsible se cobrará el mayor tributo en forma de defunciones y discapacidad 1. Los datos científicos actuales indican que cuatro ti-pos de enfermedades no transmisibles (enfermedades cardiovasculares, cánceres, enfermedades respiratorias crónicas y diabetes) son las principales causas de morta-lidad en la mayor parte de los países de ingresos bajos y medianos, y exigen la adopción de medidas coordinadas. Estas enfermedades son prevenibles en gran medida mediante intervenciones eficaces que abordan los facto-res de riesgo comunes, a saber, consumo de tabaco, dieta malsana, inactividad física y uso nocivo del alcohol. Además, las mejoras del tratamiento de esas enfermeda-des pueden reducir la morbilidad, la discapacidad y la mortalidad, y contribuir a mejorar los resultados sanita-rios 2. En el año 2000, de las 3 537 000 muertes registradas en Latinoamérica y el Caribe, 67 % fueron causadas por estos padecimientos crónicos. La enfermedad isquémica del corazón y el cáncer fueron causas de la mayoría de las muertes entre las edades de 20 y 50 años. Las enfer-medades crónicas no trasmisibles (ECNT) contribuyeron a la carga global de enfermedad, con 76 % de los años de vida ajustados a la discapacidad. (AVAD). Las ECNT, además de provocar una mortalidad precoz, causan complicaciones, secuelas e invalidez, que afectan la capacidad funcional y limitan la productividad 3. Las enfermedades no trasmisibles son enfermedades de larga duración y, por lo general, de progresión lenta. Las enfermedades cardíacas, los infartos, el cáncer, las enfermedades respiratorias y la diabetes, son las princi-pales causas de mortalidad en el mundo, siendo respon-sables del 60 % de las muertes. En el 2005, 35 millones de personas murieron de una enfermedad no transmisi-ble, de las cuales la mitad era de sexo femenino y de menos de 70 años de edad 3. Se ha proyectado que para el 2020, las ENT explica-rán el 75 % de todas las muertes en el mundo, y que el
author	Rabelo Padua}, Gladys and Jacobo, Waldo and Piñera, Díaz and Gladys, Msc and Padua, Rabelo
file	:C\:/Users/Unibague/AppData/Local/Mendeley Ltd./Mendeley Desktop/Downloaded/Rabelo Padua et al. - 2012 - Enfermedades no transmisibles.

	Tendencias actuales.pdf:pdf
journal	revsaludtrabajo.sld.cu
keywords	()
number	2
pages	50
title	Enfermedades no transmisibles. Tendencias actuales
url	https://revsaludtrabajo.sld.cu/index.php/revsyt/article/view/609
volume	13
year	2012
**article**	**Tang2019a**
abstract	At present, deep learning has been widely adopted in medical image processing. However, the current deep neural networks depend on a large number of labeled training data, but medical images segmentation tasks often suffer from the problem of small quantity of labeled data because labeling medical images is a very expensive and time-consuming task. In order to overcome this difficulty, this paper proposes a new image augmentation strategy based on statistical shape model and three-dimensional thin plate spline, which can generate many simulated images from a small number of real images. Firstly, the shape information of the real labeled images is modeled with the statistical shape model, and a series of simulated shapes are generated by sampling from this model. Secondly, the simulated shapes are filled with texture using three-dimensional thin plate spline to generate the simulated images. Finally, the simulated images and the real images are used together for training deep neural networks. The proposed framework is a general data augmentation method that can be used in any anatomical structure segmentation tasks with any deep neural network architecture. We used two different datasets, including prostate MRI dataset and liver CT dataset, and used two different deep network structures, including multi-scale 3D Convolutional Neural Networks (multi-scale 3D CNN) and U-net. The experimental results showed that the proposed data augmentation strategy can improve the accuracy of existing segmentation algorithms based on deep neural networks.
author	Tang, Zhixian and Chen, Kun and Pan, Mingyuan and Wang, Manning and Song, Zhijian
doi	10.1109/ACCESS.2019.2941154
file	:C\:/Users/Unibague/AppData/Local/Mendeley Ltd./Mendeley Desktop/Downloaded/Tang et al. - 2019 - An Augmentation Strategy for Medical Image Processing Based on Statistical Shape Model and 3D Thin Plate Spline for.pdf:pdf
issn	21693536
journal	IEEE Access

keywords	3D thin plate spline,Augmentation strategy,deep learning,image segmentation,statistical shape model
pages	133111--133121
publisher	Institute of Electrical and Electronics Engineers Inc.
title	An Augmentation Strategy for Medical Image Processing Based on Statistical Shape Model and 3D Thin Plate Spline for Deep Learning
volume	7
year	2019
**article**	**Huang2024**
abstract	Cardiovascular disease (CVD) remains the leading cause of death worldwide. Assessing of CVD risk plays an essential role in identifying individuals at higher risk and enables the implementation of targeted intervention strategies, leading to improved CVD prevalence reduction and patient survival rates. The ocular vasculature, particularly the retinal vasculature, has emerged as a potential means for CVD risk stratification due to its anatomical similarities and physiological characteristics shared with other vital organs, such as the brain and heart. The integration of artificial intelligence (AI) into ocular imaging has the potential to overcome limitations associated with traditional semi-automated image analysis, including inefficiency and manual measurement errors. Furthermore, AI techniques may uncover novel and subtle features that contribute to the identification of ocular biomarkers associated with CVD. This review provides a comprehensive overview of advancements made in AI-based ocular image analysis for predicting CVD, including the prediction of CVD risk factors, the replacement of traditional CVD biomarkers (e.g., CT-scan measured coronary artery calcium score), and the prediction of symptomatic CVD events. The review covers a range of ocular imaging modalities, including colour fundus photography, optical coherence tomography, and optical coherence tomography angiography, and other types of images like external eye images. Additionally, the review addresses the current limitations of AI research in this field and discusses the challenges associated with translating AI algorithms into clinical practice.
author	Huang, Yu and Cheung, Carol Y. and Li, Dawei and Tham, Yih Chung and Sheng, Bin and Cheng, Ching Yu and Wang, Ya Xing and Wong, Tien Yin
doi	10.1038/S41433-023-02724-4
issn	14765454
journal	Eye (Basingstoke)
month	feb
number	3
pages	464--472

pmid	37709926
publisher	Springer Nature
title	AI-integrated ocular imaging for predicting cardiovascular disease: advancements and future outlook
volume	38
year	2024
**article**	**Wong2020**
abstract	Artificial intelligence (AI) is becoming a vital concept in medicine leading to a rapid emergence of important tools for medical diagnostics. Now, as a crucial machine learning tool in the field of computer vision, deep learning (DL) is being widely used in medical imaging. Furthermore, as reported in the medical literature, DL has been widely used in medical related research. However, the practical application of DL in clinical diagnosis is relatively small, and it is a new field that may have some challenges. How to effectively perform medical image analysis is a major problem in the field of disease diagnosis, and further diagnostic methods need to be developed. At this stage, DL could be viewed as a black box requiring knowledge of its internal workings, and hence presents some crucial technical challenges that need further methodological development. Thereafter with proper diagnostics, pre-operative computerized simulation planning can be carried out for use of appropriate surgical intervention technology. This paper presents important questions on cardiovascular disease (CVD) diagnostics, using this powerful and yet not adequately understood technology. It discusses issues brought by the paradigm shift of AI vis-à-vis DL in CVD diagnostics, provides possible solutions to potential issues, and envisions the future of the related machine intelligence applications. The discussed problems are dissected into the modular aspects of DL in relation to CVD image classification, segmentation, and detection. A proper perspective on management of these issues is the key to a successful technological implementation of DL in modern medical science.
author	Wong, Kelvin K.L. and Fortino, Giancarlo and Abbott, Derek
doi	10.1016/j.future.2019.09.047
issn	0167739X
journal	Future Generation Computer Systems
number	xxxx
pages	802--811
publisher	Elsevier B.V.
title	Deep learning-based cardiovascular image diagnosis: A promising challenge
volume	110

year	2020
**article**	**Dhingra2020a**
abstract	Digital technology has been a revolutionary foray in education, industry, research and recently, healthcare. Digital health encompasses various aspects of technology like information and communication, mobile health, data-recording and telemedicine. There has been an exponential and unregulated increase in digital health services in last few years which have raised concerns over data privacy, ethical standards and quality of services. The World Health Organization recently released the global strategy on digital health as a visionary document that provides a framework for countries to implement and expand digital health services. The following update briefly highlights the salient features of the update.
author	Dhingra, Dhulika and Dabas, Aashima
doi	10.1007/S13312-020-1789-7
file	:C\:/Users/Unibague/AppData/Local/Mendeley Ltd./Mendeley Desktop/Downloaded/Dhingra, Dabas - 2020 - Global Strategy on Digital Health.pdf:pdf
issn	09747559
journal	Indian Pediatrics
keywords	Health system,Public health,Technology,eHealth strategy
month	apr
number	4
pages	356--358
pmid	32284477
publisher	Springer
title	Global Strategy on Digital Health
volume	57
year	2020
**article**	**Turcu2023**
abstract	The increase in the incidence of cardiovascular diseases worldwide raises concerns about the urgent need to increase definite measures for the self-determination of different parameters, especially those defining cardiac function. Heart rate variability (HRV) is a non-invasive method used to evaluate autonomic nervous system modulation on the cardiac sinus node, thus describing the oscillations between consecutive electrocardiogram

	R-R intervals. These fluctuations are undetectable except when using specialized devices, with ECG Holter monitoring considered the gold standard. HRV is considered an independent biomarker for measuring cardiovascular risk and for screening the occurrence of both acute and chronic heart diseases. Also, it can be an important predictive factor of frailty or neurocognitive disorders, like anxiety and depression. An increased HRV is correlated with rest, exercise, and good recovery, while a decreased HRV is an effect of stress or illness. Until now, ECG Holter monitoring has been considered the gold standard for determining HRV, but the recent decade has led to an accelerated development of technology using numerous devices that were created specifically for the pre-hospital self-monitoring of health statuses. The new generation of devices is based on the use of photoplethysmography, which involves the determination of blood changes at the level of blood vessels. These devices provide additional information about heart rate (HR), blood pressure (BP), peripheral oxygen saturation (SpO2), step counting, physical activity, and sleep monitoring. The most common devices that have this technique are smartwatches (used on a large scale) and chest strap monitors. Therefore, the use of technology and the self-monitoring of heart rate and heart rate variability can be an important first step in screening cardiovascular pathology and reducing the pressure on medical services in a hospital. The use of telemedicine can be an alternative, especially among elderly patients who are associated with walking disorders, frailty, or neurocognitive disorders.
author	Turcu, Ana Maria and Ilie, Adina Carmen and Ștefăniu, Ramona and Țăranu, Sabinne Marie and Sandu, Ioana Alexandra and Alexa-Stratulat, Teodora and Pîslaru, Anca Iuliana and Alexa, Ioana Dana
doi	10.3390/diagnostics13142382
issn	20754418
journal	Diagnostics
number	14
pages	1--11
title	The Impact of Heart Rate Variability Monitoring on Preventing Severe Cardiovascular Events
volume	13
year	2023
**article**	**Huang2023**
abstract	Cardiovascular disease (CVD) remains the leading cause of death worldwide. Assessing of CVD risk plays an essential role in identifying individuals at higher risk and enables the implementation of targeted intervention strategies, leading to improved CVD prevalence reduction and patient survival rates. The ocular vasculature, particularly the retinal vasculature, has emerged as a potential means for CVD risk stratification due to its anatomical similarities and physiological characteristics shared with other vital organs, such as the brain and heart. The integration of artificial intelligence (AI) into ocular imaging has the potential to overcome limitations associated with traditional semi-automated image analysis, including inefficiency and manual measurement errors. Furthermore, AI techniques may uncover novel and subtle features that contribute to the identifica-

	tion of ocular biomarkers associated with CVD. This review provides a comprehensive overview of advancements made in AI-based ocular image analysis for predicting CVD, including the prediction of CVD risk factors, the replacement of traditional CVD biomarkers (e.g., CT-scan measured coronary artery calcium score), and the prediction of symptomatic CVD events. The review covers a range of ocular imaging modalities, including colour fundus photography, optical coherence tomography, and optical coherence tomography angiography, and other types of images like external eye images. Additionally, the review addresses the current limitations of AI research in this field and discusses the challenges associated with translating AI algorithms into clinical practice.
author	Huang, Yu and Cheung, Carol Y. and Li, Dawei and Tham, Yih Chung and Sheng, Bin and Cheng, Ching Yu and Wang, Ya Xing and Wong, Tien Yin
doi	10.1038/s41433-023-02724-4
issn	14765454
journal	Eye (Basingstoke)
number	September
title	AI-integrated ocular imaging for predicting cardiovascular disease: advancements and future outlook
year	2023
**article**	**Yeung2022a**
abstract	Background: Digital technology uses in cardiology have become a popular research focus in recent years. However, there has been no published bibliometric report that analyzed the corresponding academic literature in order to derive key publishing trends and characteristics of this scientific area. Objective: We used a bibliometric approach to identify and analyze the academic literature on digital technology uses in cardiology, and to unveil popular research topics, key authors, institutions, countries, and journals. We further captured the cardiovascular conditions and diagnostic tools most commonly investigated within this field. Methods: The Web of Science electronic database was queried to identify relevant papers on digital technology uses in cardiology. Publication and citation data were acquired directly from the database. Complete bibliographic data were exported to VOSviewer, a dedicated bibliometric software package, and related to the semantic content of titles, abstracts, and keywords. A term map was constructed for findings visualization. Results: The analysis was based on data from 12,529 papers. Of the top 5 most productive institutions, 4 were based in the United States. The United States was the most productive country (4224/12,529, 33.7%), followed by United Kingdom (1136/12,529, 9.1%), Germany (1067/12,529, 8.5%), China (682/12,529, 5.4%), and Italy (622/12,529, 5.0%). Cardiovascular diseases that had been frequently investigated included hypertension (152/12,529, 1.2%), atrial fibrillation (122/12,529, 1.0%), atherosclerosis (116/12,529, 0.9%), heart failure (106/12,529, 0.8%), and arterial stiffness (80/12,529, 0.6%). Recurring modalities were electrocardiography (170/12,529, 1.4%), angiography (127/12,529, 1.0%), echocardiography (127/12,529, 1.0%), digital subtraction angiography (111/12,529, 0.9%), and photoplethysmography (80/12,529, 0.6%). For a literature subset on smartphone apps and wearable devices, the Journal of Medical Internet Research (20/632, 3.2%) and other JMIR portfolio journals (51/632, 8.0%) were the major publishing venues. Conclusions: Digital technology uses in cardiology tar-

	get physicians, patients, and the general public. Their functions range from assisting diagnosis, recording cardiovascular parameters, and patient education, to teaching laypersons about cardiopulmonary resuscitation. This field already has had a great impact in health care, and we anticipate continued growth.
author	Yeung, Andy Wai Kan and Kulnik, Stefan Tino and Parvanov, Emil D. and Fassl, Anna and Eibensteiner, Fabian and Völkl-Kernstock, Sabine and Kletecka-Pulker, Maria and Crutzen, Rik and Gutenberg, Johanna and Höppchen, Isabel and Niebauer, Josef and Smeddinck, Jan David and Willschke, Harald and Atanasov, Atanas G.
doi	10.2196/36086
issn	14388871
journal	Journal of Medical Internet Research
keywords	atrial fibrillation,cardiac,cardiology,cardiopulmonary resuscitation,cardiovascular,electrocardiography,health application,heart,hypertension,photoplethysmography,wearable device, digital health, mHealth
month	may
number	5
pmid	35544307
publisher	JMIR Publications Inc.
title	Research on Digital Technology Use in Cardiology: Bibliometric Analysis
volume	24
year	2022
**article**	**Javaid2022**
abstract	Machine learning (ML) refers to computational algorithms that iteratively improve their ability to recognize patterns in data. The digitization of our healthcare infrastructure is generating an abundance of data from electronic health records, imaging, wearables, and sensors that can be analyzed by ML algorithms to generate personalized risk assessments and promote guideline-directed medical management. ML's strength in generating insights from complex medical data to guide clinical decisions must be balanced with the potential to adversely affect patient privacy, safety, health equity, and clinical interpretability. This review provides a primer on key advances in ML for cardiovascular disease prevention and how they may impact clinical practice.
author	Javaid, Aamir and Zghyer, Fawzi and Kim, Chang and Spaulding, Erin M. and Isakadze, Nino and Ding, Jie and Kargillis, Daniel and Gao, Yumin and Rahman, Faisal and Brown, Donald E. and Saria, Suchi and Martin, Seth S. and Kramer, Christopher M. and Blumenthal, Roger S. and Mar-

	vel, Francoise A.
doi	10.1016/j.ajpc.2022.100379
issn	26666677
journal	American Journal of Preventive Cardiology
number	July
pages	100379
publisher	Elsevier B.V.
title	Medicine 2032: The future of cardiovascular disease prevention with machine learning and digital health technology
volume	12
year	2022
**article**	**Azmi2022**
abstract	There is a considerable rise in cardiovascular diseases in the world. It is pertinently essential to make cardiovascular prediction accurate to the maximum. A forecast based on machine learning techniques can be beneficial in detecting cardiovascular disease (CVD) with maximum precision and accuracy. The disease's effective prediction helps in early diagnosis, which cuts down the mortality rate. A health history and the causes of heart disease require the efficient detection and prediction of CVD. Data analytics is beneficial for making predictions based on a massive amount of data, and it aids health clinics in disease prognosis. Regularly, a large volume of patient-related data is maintained. The information gathered can be used to forecast the emergence of upcoming diseases. Our study presents a detailed comparative study of Cardiovascular Disease by comparing the various machine learning techniques mainly comprising of classification and predictive algorithms. The study shows an in-depth analysis of around forty-one papers related to cardiovascular disease by using machine learning techniques. This study evaluates the selected publications rigorously and identifies gaps in the available literature, making it useful for researchers to develop and apply in clinical fields, primarily on datasets related to heart disease. The current study will aid medical practitioners in predicting heart threats ahead of time, allowing them to take preventative measures.
author	Azmi, Javed and Arif, Muhammad and Nafis, Md Tabrez and Alam, M. Afshar and Tanweer, Safdar and Wang, Guojun
doi	10.1016/j.medengphy.2022.103825
issn	18734030
journal	Medical Engineering and Physics
title	A systematic review on machine learning approaches for cardiovascular disease prediction using medical big data

volume	105
year	2022
**article**	**Turcu2023b**
abstract	Citation: Turcu, A.-M.; Ilie, A.C.; S , tef˘ aniu, R.; T , ˘ aranu, S.M.; Sandu, I.A.; Alexa-Stratulat, T.; Pîslaru, A.I.; Alexa, I.D. The Impact of Heart Rate Variability Monitoring on Preventing Severe Cardiovascular Events. Diagnostics 2023, 13, 2382. https:// Abstract: The increase in the incidence of cardiovascular diseases worldwide raises concerns about the urgent need to increase definite measures for the self-determination of different parameters, especially those defining cardiac function. Heart rate variability (HRV) is a non-invasive method used to evaluate autonomic nervous system modulation on the cardiac sinus node, thus describing the oscillations between consecutive electrocardiogram R-R intervals. These fluctuations are undetectable except when using specialized devices, with ECG Holter monitoring considered the gold standard. HRV is considered an independent biomarker for measuring cardiovascular risk and for screening the occurrence of both acute and chronic heart diseases. Also, it can be an important predictive factor of frailty or neurocognitive disorders, like anxiety and depression. An increased HRV is correlated with rest, exercise, and good recovery, while a decreased HRV is an effect of stress or illness. Until now, ECG Holter monitoring has been considered the gold standard for determining HRV, but the recent decade has led to an accelerated development of technology using numerous devices that were created specifically for the pre-hospital self-monitoring of health statuses. The new generation of devices is based on the use of photoplethysmography, which involves the determination of blood changes at the level of blood vessels. These devices provide additional information about heart rate (HR), blood pressure (BP), peripheral oxygen saturation (SpO 2), step counting, physical activity, and sleep monitoring. The most common devices that have this technique are smartwatches (used on a large scale) and chest strap monitors. Therefore, the use of technology and the self-monitoring of heart rate and heart rate variability can be an important first step in screening cardiovascular pathology and reducing the pressure on medical services in a hospital. The use of telemedicine can be an alternative, especially among elderly patients who are associated with walking disorders, frailty, or neurocognitive disorders.
author	Turcu, Ana-Maria and {Carmen Ilie}, Adina and Marie, Sabinne T and {Alexandra Sandu}, Ioana and Alexa-Stratulat, Teodora and {Iuliana Pîslaru}, Anca and {Dana Alexa}, Ioana
doi	10.3390/diagnostics13142382
file	:C\:/Users/Unibague/AppData/Local/Mendeley Ltd./Mendeley Desktop/Downloaded/Turcu et al. - 2023 - The impact of heart rate variability monitoring on preventing severe cardiovascular events.pdf:pdf
journal	mdpi.comAM Turcu, AC Ilie, R Ștefăniu, SM Țăranu, IA Sandu, T Alexa-Stratulat, AI Pîslaru, ID AlexaDiagnostics, 2023•mdpi.com
keywords	heart rate variability,novel wearable,wearable devices
month	jul
number	14

publisher	Multidisciplinary Digital Publishing Institute (MDPI)
title	The impact of heart rate variability monitoring on preventing severe cardiovascular events
url	https://www.mdpi.com/2075-4418/13/14/2382
volume	13
year	2023
**article**	**Lindquist2021**
abstract	With the rate of cardiovascular diseases in the U.S increasing throughout the years, there is a need for developing more advanced treatment plans that can be tailored to specific patients and scenarios. The development of 3D printing is rapidly gaining acceptance into clinical cardiology. In this review, key technologies used in 3D printing are briefly summarized, particularly, the use of artificial intelligence (AI), open-source tools like MeshLab and MeshMixer, and 3D printing techniques such as fused deposition molding (FDM) and polyjet are reviewed. The combination of 3D printing, multiple image integration, and augmented reality may greatly enhance data visualization during diagnosis, treatment planning, and surgical procedures for cardiology.
author	Lindquist, Ellen M. and Gosnell, Jordan M. and Khan, Sana K. and Byl, John L. and Zhou, Weihua and Jiang, Jingfeng and Vettukattil, Joseph J.
doi	10.1016/j.stlm.2021.100034
issn	26669641
journal	Annals of 3D Printed Medicine
pages	100034
publisher	Elsevier Masson SAS
title	3D printing in cardiology: A reLindquist, E. M., Gosnell, J. M., Khan, S. K., Byl, J. L., Zhou, W., Jiang, J., & Vettukattil, J. J. (2021). 3D printing in cardiology: A review of applications and roles for advanced cardiac imaging. Annals of 3D Printed Me
volume	4
year	2021
**article**	**Yin2019a**
abstract	Background: Digital health is poised to transform health care and redefine personalized health. As Internet and mobile phone usage increases, as technology develops new ways to collect data, and as clinical guidelines change, all areas of medicine face new challenges and opportunities. Inflammatory bowel disease (IBD) is one of many chronic diseases that may benefit from these advances in digital health. This review intends to lay a foundation for clinicians and technologists to understand future directions and opportunities together. Objective: This review covers digital health

	apps that have been used in IBD, how they have fit into a clinical care framework, and the challenges that clinicians and technologists face in approaching future opportunities. Methods: We searched PubMed, Scopus, and ClinicalTrials.gov to identify digital health apps that have been studied and were published in the literature from January 1, 2010, to April 19, 2019. The search terms were "mobile health" OR "eHealth" OR "digital health" OR "smart phone" OR "mobile app" OR "mobile applications" OR "mHealth" OR "smartphones" AND "IBD" OR "Inflammatory bowel disease" OR "Crohn's Disease" (CD) OR "Ulcerative Colitis" (UC) OR "UC" OR "CD," followed by further analysis of citations from the results. We searched the Apple iTunes app store to identify a limited selection of commercial apps to include for discussion. Results: A total of 68 articles met the inclusion criteria. A total of 11 digital health apps were identified in the literature and 4 commercial apps were selected to be described in this review. While most apps have some educational component, the majority of apps focus on eliciting patient-reported outcomes related to disease activity, and a few are for treatment management. Significant benefits have been seen in trials relating to education, quality of life, quality of care, treatment adherence, and medication management. No studies have reported a negative impact on any of the above. There are mixed results in terms of effects on office visits and follow-up. Conclusions: While studies have shown that digital health can fit into, complement, and improve the standard clinical care of patients with IBD, there is a need for further validation and improvement, from both a clinical and patient perspective. Exploring new research methods, like microrandomized trials, may allow for more implementation of technology and rapid advancement of knowledge. New technologies that can objectively and seamlessly capture remote data, as well as complement the clinical shift from symptom-based to inflammation-based care, will help the clinical and health technology communities to understand the full potential of digital health in the care of IBD and other chronic illnesses.
author	Yin, Andrew Lukas and Hachuel, David and Pollak, John P. and Scherl, Ellen J. and Estrin, Deborah
doi	10.2196/14630
issn	14388871
journal	Journal of Medical Internet Research
keywords	Crohn's disease,digital health,eHealth,inflammatory bowel disease,mHealth,mobile health,mobile technology,review,ulcerative colitis
month	aug
number	8
pmid	31429410
publisher	JMIR Publications Inc.
title	Digital health apps in the clinical care of inflammatory bowel disease: Scoping review
volume	21
year	2019

article	Visualization2021
abstract	The article presents the continuation of the author's research in the field of visualization of biomedical signals in the development and operation of mobile cardiodiagnostic systems. Some of the latest guidelines for developers of electrocardiographic equipment and recommendations for the frequency range of the ECG signal for standard clinical use are considered. The negative impact of industrial frequency interference (power supply network interference) on the analyzed signal is emphasized. The hardware and software solutions of means of removal and registration of interference of the power supply network are offered, including a laryngophone headset with a small-sized condenser digital USB microphone and a sound editor. During the period 2010-2020, 1092 phonograms of acoustic biomedical heart signals, network interference and electromagnetic interference in various rooms were recorded. To solve the problems of visualization of power supply network interference, the capabilities of the common audio editor Audacity were evaluated. The necessary time-frequency resolution was not achieved using the examples of the obtained spectral sections of the signal sections with interference. The developed software tools WaveView and WaveView-MWA are presented, which provide high frequency-time resolution and visual visualization of small-level interference due to multi-level wavelet analysis of non-stationary signals. Experimental testing of the proposed visualization technology has confirmed its high efficiency. For the first time, a set of typical power supply network interference signals was obtained when registering biomedical cardiodiagnostic signals under real operating conditions of mobile systems. An example of noise visualization using the portal is given acustocard.ru in on-line mode.
author	Visualization, Y Gorshkov - Scientific and undefined 2021
doi	10.26583/sv.13.1.04
file	:C\:/Users/Unibague/AppData/Local/Mendeley Ltd./Mendeley Desktop/Downloaded/Visualization, 2021 - 2021 - Visualization of power supply network interference in telemedicine systems of mobile electrocardiography.pdf:pdf
journal	sv-journal.orgY GorshkovScientific Visualization, 2021•sv-journal.org
keywords	mobile electrocardiography,multi-level wavelet analysis,power supply network interference,telemedicine systems
number	1
pages	44--53
title	Visualization of power supply network interference in telemedicine systems of mobile electrocardiography
url	http://sv-journal.org/2021-1/04/en.pdf
volume	13
year	2021
article	Briganti2020
abstract	Artificial intelligence-powered medical technologies are rapidly evolving into applicable solutions for clinical practice. Deep learning algorithms

	can deal with increasing amounts of data provided by wearables, smartphones, and other mobile monitoring sensors in different areas of medicine. Currently, only very specific settings in clinical practice benefit from the application of artificial intelligence, such as the detection of atrial fibrillation, epilepsy seizures, and hypoglycemia, or the diagnosis of disease based on histopathological examination or medical imaging. The implementation of augmented medicine is long-awaited by patients because it allows for a greater autonomy and a more personalized treatment, however, it is met with resistance from physicians which were not prepared for such an evolution of clinical practice. This phenomenon also creates the need to validate these modern tools with traditional clinical trials, debate the educational upgrade of the medical curriculum in light of digital medicine as well as ethical consideration of the ongoing connected monitoring. The aim of this paper is to discuss recent scientific literature and provide a perspective on the benefits, future opportunities and risks of established artificial intelligence applications in clinical practice on physicians, healthcare institutions, medical education, and bioethics.
author	Briganti, Giovanni and {Le Moine}, Olivier
doi	10.3389/fmed.2020.00027
issn	2296858X
journal	Frontiers in Medicine
number	February
pages	1--6
title	Artificial Intelligence in Medicine: Today and Tomorrow
volume	7
year	2020
**article**	**Rubis-Prat2005**
abstract	Cardiovascular diseases are the leading cause of death worldwide; more people die each year from cardiovascular disease than from any other cause of death. In Costa Rica, cardiovascular diseases suffer from one of the most common causes in the mortality rate. Cardiovascular risk is defined as the probability of suffering a cardiovascular event in a given period. On the other hand, cardiovascular risk factors are those biological signs or acquired habits that occur most frequently in patients with any cardiovascular disease (arterial hypertension, coronary heart disease, cerebrovascular disease, among others). Cardiovascular diseases have a multifactorial origin; therefore, a risk factor must be considered in the context of others. Cardiovascular risk factors are divided into 2 large groups: non- modifiable such as age, sex and family history, and modifiable, including hypercholesterolemia, smoking, diabetes, high blood pressure, obesity and sedentary lifestyle.
author	Rubis-Prat, J.
doi	10.1016/s0211-3449(05)73753-x

issn	03045412
journal	Medicine - Programa de Formaci?n M?dica Continuada Acreditado
number	38
pages	2506--2513
title	Factores de riesgo cardiovascular
volume	9
year	2005
**article**	**KERR1952**
author	KERR, W. J. and LAGEN, J. B.
journal	Archivos médicos de Cuba
number	4
pages	413--416
pmid	14953784
title	Enfermedades cardiovasculares.
volume	3
year	1952
**article**	**Zheng2022**
author	Zheng, Zhonghui and Zhang, Ping and Yuan, Fangzheng and Bo, Yunque
issn	1660-4601
journal	International Journal of Environmental Research and Public Health
number	9
pages	5625
publisher	MDPI
title	Scientometric analysis of the relationship between a built environment and cardiovascular disease

volume	19
year	2022
**article**	**Freak-Poli2023**
author	Freak-Poli, Rosanne and Hu, Jessie and Phyo, Aung Zaw Zaw and Barker, S Fiona
issn	1660-4601
journal	International Journal of Environmental Research and Public Health
number	6
pages	4853
publisher	MDPI
title	Social isolation and social support influence health service utilisation and survival after a cardiovascular disease event: a systematic review
volume	20
year	2023
**article**	**Gauffriau2007**
author	Gauffriau, Marianne and Larsen, Peder Olesen and Maye, Isabelle and Roulin-Perriard, Anne and von Ins, Markus
issn	0138-9130
journal	Scientometrics
pages	175--214
publisher	Springer
title	Publication, cooperation and productivity measures in scientific research
volume	73
year	2007
**article**	**Chou2023**
author	Chou, Hsiang-Yun and Tsai, Ya-Wen and Ma, Shang-Chun and Ma, Shang-Min and Shih, Chia-Li and Yeh, Chieh-Ting
issn	1660-4601

journal	International journal of environmental research and public health
number	2
pages	1419
publisher	MDPI
title	Efficacy and cost over 12 hospitalization weeks of postacute care for stroke
volume	20
year	2023
**article**	**Attaran2022**
author	Attaran, Mohsen
issn	2047-9700
journal	International Journal of Healthcare Management
number	1
pages	70--83
publisher	Taylor & Francis
title	Blockchain technology in healthcare: Challenges and opportunities
volume	15
year	2022
**article**	**Weismayer2017**
author	Weismayer, Christian and Pezenka, Ilona
issn	0138-9130
journal	Scientometrics
number	3
pages	1757--1785
publisher	Springer

title	Identifying emerging research fields: a longitudinal latent semantic keyword analysis
volume	113
year	2017
**article**	**Wongvibulsin2019**
author	Wongvibulsin, Shannon and Martin, Seth S and Steinhubl, Steven R and Muse, Evan D
issn	1092-8464
journal	Current treatment options in cardiovascular medicine
pages	1--15
publisher	Springer
title	Connected health technology for cardiovascular disease prevention and management
volume	21
year	2019
**incollec-tion**	**France2022**
abstract	In the healthcare system, medical images are playing a vital role to identify the symptoms of early diseases by using image patterns. In the past few decades, because of increasing advancements in the healthcare systems, it produces a large volume of imaging data with different modalities (MRI, fMRI, tomosynthesis, x-ray, computed tomography, etc.) and different dimensionalities like 2D, 3D, and 4D. So, there is a necessity to develop machine learning (ML) tools to manage these healthcare data. In the medical image diagnosing process, these ML tools are used to automatically identify the different disease patterns that appear from the various modalities of medical images. One of the key challenges in medical image diagnosis using ML tools is representing a medical image in the semantic space, and extracting effective features is the crucial step; it is called the semantic gap. In the past 20 years, there are a lot of enhancements in ML techniques to reduce the semantic gap in the diagnosis of medical images. Deep learning (DL) is one of the predominant techniques, which is extensively used to reduce the semantic gap in medical image diagnosis. DL is subset of Artificial Neural Network (ANN). It has many hidden layers to learn complex patterns with different stages of abstraction. Also, in the medical image diagnosing process, DL algorithms give more accurate result than a radiologist. Particularly, the Convolutional Neural Network (CNN) was developed to study medical images. This paper gives an overview of various DL algorithms used in medical image diagnosis. We aimed to provide the key reach areas, like classification of medical images, segmentation, disease localization, and image retrieval. This will help the researcher to identify the emerging trends, research obstacles, and possible future directions in medical image diagnosis.
author	France, K. and Jaya, A. and Tiliute, Doru

booktitle	Handbook of Intelligent Healthcare Analytics: Knowledge Engineering with Big Data Analytics
doi	10.1002/9781119792550.ch11
isbn	9781119792550
keywords	Deep learning,Disease detection,Healthcare,Machine learning,Medical image analysis,Medical image retrieval,Supervised learning,Unsupervised learning
pages	233--253
publisher	Wiley Online Library
title	Machine and Deep Learning Algorithms for Healthcare Applications
volume	11
year	2022
**article**	**Lindquist2021a**
author	Lindquist, Ellen M and Gosnell, Jordan M and Khan, Sana K and Byl, John L and Zhou, Weihua and Jiang, Jingfeng and Vettukattil, Joseph J
issn	2666-9641
journal	Annals of 3D Printed Medicine
pages	100034
publisher	Elsevier
title	3D printing in cardiology: a review of applications and roles for advanced cardiac imaging
volume	4
year	2021
**article**	**Jaen-Extremera2023**
author	Jaen-Extremera, Jesus and Afanador-Restrepo, Diego Fernando and Rivas-Campo, Yulieth and Gomez-Rodas, Alejandro and Aibar-Almazan, Agustin and Hita-Contreras, Fidel and Carcelen-Fraile, Maria del Carmen and Castellote-Caballero, Yolanda and Ortiz-Quesada, Raul
issn	2077-0383
journal	Journal of clinical medicine
number	3

pages	841
publisher	MDPI
title	Effectiveness of telemedicine for reducing cardiovascular risk: a systematic review and meta-analysis
volume	12
year	2023
**article**	**Blanco-Colio2021**
abstract	Citation: Blanco-Colio, L.M.; Méndez-Barbero, N.; Pello Lázaro, A.M.; Aceña, Á.; Tarín, N.; Cristóbal, C.; Martínez-Milla, J.; González-Lorenzo, Ó.; Martín-Ventura, J.L.; Huelmos, A.; et al. MCP-1 Predicts Recurrent Cardiovascular Events in Patients with Persistent Inflammation. Abstract: Clinical data indicate that patients with C-reactive protein (CRP) levels higher than 2 mg per liter suffer from persistent inflammation, which is associated with high risk of cardio-vascular disease (CVD). We determined whether a panel of biomarkers associated with CVD could predict recurrent events in patients with low or persistent inflammation and coronary artery disease (CAD). We followed 917 patients with CAD (median 4.59 ± 2.39 years), assessing CRP, galectin-3, monocyte chemoattractant protein-1 (MCP-1), N-terminal fragment of brain natriuretic peptide (NT-proBNP) and troponin-I plasma levels. The primary outcome was the combination of cardiovas-cular events (acute coronary syndrome, stroke or transient ischemic event, heart failure or death). Patients with persistent inflammation (n = 343) showed higher NT-proBNP and MCP-1 plasma levels compared to patients with CRP < 2 mg/L. Neither MCP-1 nor NT-proBNP was associated with primary outcome in patients with CRP < 2 mg/L. However, NT-proBNP and MCP-1 plasma levels were associated with increased risk of the primary outcome in patients with persistent inflammation. When patients were divided by type of event, MCP-1 was associated with an increased risk of acute ischemic events. A significant interaction between MCP-1 and persistent inflammation was found (synergy index: 6.17 (4.39-7.95)). In conclusion, MCP-1 plasma concentration is associated with recurrent cardiovascular events in patients with persistent inflammation.
author	Blanco-Colio, Luis M and Méndez-Barbero, Nerea and María, Ana and Lázaro, Pello and Aceña, Álvaro and Tarín, Nieves and Cristóbal, Carmen and Martínez-Milla, Juan and González-Lorenzo, Óscar and {Luis Martín-Ventura}, José and Huelmos, Ana and Gutiérrez-Landaluce, Carlos and López-Castillo, Marta and Kallmeyer, Andrea and Cánovas, Ester and Alonso, Joaquín and {López Bescós}, Lorenzo and Egido, Jesús and Lorenzo, Óscar and Tuñón, José
doi	10.3390/jcm10051137
file	:C\:/Users/Unibague/AppData/Local/Mendeley Ltd./Mendeley Desktop/Downloaded/Blanco-Colio et al. - 2021 - MCP-1 predicts recurrent cardiovascular events in patients with persistent inflammation.pdf:pdf
journal	mdpi.com
keywords	C-reactive protein,MCP-1,NT-proBNP,inflammation
pages	1137

title	MCP-1 predicts recurrent cardiovascular events in patients with persistent inflammation
url	https://www.mdpi.com/2077-0383/10/5/1137
volume	10
year	2021
